# SMRT- and Illumina-based RNA-seq analyses unveil the ginsinoside biosynthesis and transcriptomic complexity in *Panax notoginseng*

**DOI:** 10.1038/s41598-020-72291-1

**Published:** 2020-09-17

**Authors:** Dan Zhang, Wei Li, Zhong-jian Chen, Fu-gang Wei, Yun-long Liu, Li-zhi Gao

**Affiliations:** 1 Institution of Genomics and Bioinformatics, South China Agricultural University, Guangzhou, 510642 China; 2 Wenshan Sanqi Institute of Science and Technology, Wenshan University, Wenshan, 663000 China; 3 Wenshan Miaoxiang Notoginseng Industral Co., LTD, Wenshan, 663000 China; 4 Plant Germplasm and Genomics Center, Kunming Institute of Botany, The Chinese Academy of Sciences, Kunming, 650204 China

**Keywords:** Gene regulation, Transcriptomics

## Abstract

*Panax notoginseng* is one of the most widely used traditional Chinese herbs with particularly valued roots. Triterpenoid saponins are mainly specialized secondary metabolites, which medically act as bioactive components. Knowledge of the ginsenoside biosynthesis in *P. notoginseng*, which is of great importance in the industrial biosynthesis and genetic breeding program, remains largely undetermined. Here we combined single molecular real time (SMRT) and Second-Generation Sequencing (SGS) technologies to generate a widespread transcriptome atlas of *P. notoginseng*. We mapped 2,383 full-length non-chimeric (FLNC) reads to adjacently annotated genes, corrected 1,925 mis-annotated genes and merged into 927 new genes. We identified 8,111 novel transcript isoforms that have improved the annotation of the current genome assembly, of which we found 2,664 novel lncRNAs. We characterized more alternative splicing (AS) events from SMRT reads (20,015 AS in 6,324 genes) than Illumina reads (18,498 AS in 9,550 genes), which contained a number of AS events associated with the ginsenoside biosynthesis. The comprehensive transcriptome landscape reveals that the ginsenoside biosynthesis predominantly occurs in flowers compared to leaves and roots, substantiated by levels of gene expression, which is supported by tissue-specific abundance of isoforms in flowers compared to roots and rhizomes. Comparative metabolic analyses further show that a total of 17 characteristic ginsenosides increasingly accumulated, and roots contained the most ginsenosides with variable contents, which are extraordinarily abundant in roots of the three-year old plants. We observed that roots were rich in protopanaxatriol- and protopanaxadiol-type saponins, whereas protopanaxadiol-type saponins predominated in aerial parts (leaves, stems and flowers). The obtained results will greatly enhance our understanding about the ginsenoside biosynthetic machinery in the genus *Panax*.

## Introduction

*Panax notoginseng* (Burk) F. H. Chen is a perennial herbaceous plant, belonging to the genus *Panax*, Araliaceae. It is a diploid (2n = 2X = 24) with the haploid genome size of ~ 2.0 gigabases^1–3^, while the ginseng (*P. ginseng*) and American ginseng (*P. quinquefolium*), are tetraploids (2n = 4X = 48) with haploid genome sizes of ~ 3.2 and 4.9 gigabases, respectively^4–6^. *P. notoginseng* is mainly cultivated in Yunnan Province, China, consuming as a famous traditional Chinese herb for about 400 years^7^. The dried roots of this medicinal plant are commonly used as the top class of traditional Chinese herbs for more than 2,000 years. The *P. notoginseng* has been best known for its functions in the treatment of cardiovascular diseases and valuable hemostatic effects. *P. notoginseng* pharmacologically possesses anti-thrombotic, anti-hypertensive, anti-atherosclerotic, neuroprotective and hepatoprotective activities^8^. It has also contributed principal components to Yunnan Bai Yao and Xue Sai Tong, which are worldwide renowned medicinal products for hematologic diseases. Among a number of active ingredients in the herb, such as triterpene saponins, flavonoids and polysaccharides, triterpene saponins are the most medicinally valued^9^. To date, more than 60 triterpene saponins have been isolated and characterized from different tissues of *P. notoginseng*, for example, 20 (S)-protopanaxadiol and 20 (S)-protopanaxatriol^9^. Many of these saponins are similar to *P. ginseng* and *P. quinquefolium*, but some phytochemical constituents are predominantly biosynthesized in *P. notoginseng*. It was reported that total saponins were highest in *P. notoginseng* but lowest in *P. ginseng*, while the ratio of panaxatriol and panaxadiol of ginsenosides in *P. notoginseng* (3:1) is higher than those of *P. ginseng* (1:2) and *P. quinquefolium* (1:3)^10^.

Ginsenosides are biosynthesized from terpenoid precursors, 2, 3-oxidosqualene, which is synthesized via the mevalonate (MVA) and 2-C-methyl-D-erythritol-4-phosphate (MEP) pathways; the MVA is located in cytosol while the MEP is in plastids^11^. The 2, 3-oxidosqualene is cyclized by oxidosqualene cyclases (OSCs), generating the two specific triterpenoid backbones (dammarenediol-II and β-amyrin); these *OSC* genes include dammarenediol synthase (*DDS*) and β-amyrin (*AS*)^12–14^. Triterpenoids are then modified by some specific cytochrome P450-dependent monoxygenases (*CYP450s*) and UDP-dependent glycosyltransferases (*UGTs*), resulting in a number of specific ginsenosides^15^. Ginsenosides are mainly derived from natural products, which are fairly difficult to synthesize in vitro, largely limiting industry production of ginsenosides. Whereas, *P. notoginseng* is a perennial plant, which has long suffered from serious cultivation problems, such as serious diseases and successive cropping obstacle, largely decreasing the quality and yields of *P. notoginseng* and ginsenosides as well^16,17^. Therefore, the genetic manipulation of secondary metabolic pathways and genetic improvement program of *P. notoginseng* are the two efficient strategies to ensure the ginsenoside production. With this regard, a comprehensive knowledge about the ginsenoside biosynthesis is rather necessary for the efficient utilization of the *Panax* species.

The *P. notoginseng* transcriptomes were generated by taking advantage of SGS-based RNA-Seq for roots, leaves, and flowers, of which a number of genes involved in the ginsenoside biosynthesis were preliminarily identified^18,19^. The completion of the first high-quality genome assembly of *P. notoginseng* by SGS platforms has further provided novel insights into the ginsenoside biosynthesis^3^. Comparative transcriptome analyses recently showed that three triterpene saponins (R1, Rb1, and Rg1) were highly accumulated in the roots of 3-year-old plants than 1-year-old plants of *P. notoginseng*, and most genes involved in the saponin biosynthesis increased in roots during the growth periods^20^. One more transcriptome analysis reported that the activated TCA cycle, as revealed by up-regulation of *ACLA-3* and some key metabolites in this cycle, may play an important role in the increased yield of *Panax notoginseng* roots when treated with both ammonium and nitrate fertilizers^21^. However, it is still challenging to acquire full-length cDNAs towards a well-annotated transcriptome atlas of *P. notoginseng* because the limitation of SGS-based RNA-seq technology that only generates short sequencing reads constraints to assemble long or complete transcripts. The single-molecule real-time (SMRT) sequencing, which is developed by Pacific BioSciences (PacBio), provides an alternative approach to overcome short-reads sequencing limitations, such as the assembly and determination of complex genomic regions, gene isoform discovery, and methylation detection^22^. PacBio sequencing platform offers much longer read lengths, which eliminate the need for computational assemblies of transcriptomes. This sequencing technology has been extensively applied to an increasing number of plant species, such as rice^23^, soybean^24^, *Salvia miltiorrhiza*^25^, *Sorghum bicolor*^26^, *Zea mays*^27^, *Coffea arabica*^28^, *Fragaria vesca*^29^, and *Phyllostachys edulis*^30^, *Populus*^31^, switchgrass^32^ and *Ricinus communis*^33^ to better characterize the complexity of transcriptomes but not yet in *P. notoginseng*.

In this study, we present comparative transcriptomic analyses by combining the SMRT and SGS sequencing technologies to obtain a number of transcriptomes in *P. notoginseng*. To ensure far-reaching coverage of transcript isoforms and enhance the quality of the transcriptome, we generated the data set from the five pooled tissues (rhizomes, roots, stems, leaves, flowers) of *P. notoginseng* using PacBio Iso-Seq technology. In parallel, messenger RNA generated individually from thirty-eight unpooled tissues, were separately sequenced on the Illumina HiSeq 2000 platform to support our PacBio-based analyses and quantify gene/isoform expression. Our deep comparative analyses have confirmed the power of the SMRT sequencing technology that efficiently complements short-read sequencing in accurately reconstructing full-length splice variants and discovering a large number of novel genes and a high-confidence alternatively spliced isoforms. Based on the high-quality reference transcriptome as well as rich tissue-specific expression profiles we better identified specific genes and obtained in-depth insights into metabolic pathways that will enable to elucidate the mechanisms underlying the ginsenoside biosynthesis in *P. notoginseng*. Overall, high-quality SMRT-based RNA-seq data set that has largely improved the annotation of the transcriptome and draft genome assembly of *P. notoginseng* will serve as a valuable resource to the research community.

## Results and discussion

### The SMRT and SGS sequencing, data processing and quality assessment of the *P. notoginseng* transcriptome

In order to generate high-quality reference transcriptome and comprehensive transcriptional profiles among different tissues of *P. notoginseng*, two sequencing approaches were combined using the SMRT and SGS sequencing platforms, respectively (Supplementary Fig. S1). Firstly, almost equal amount of high-quality total RNA were mixed from the five tissues, including rhizomes, roots, stems, leaves, flowers of 3-year-old plants of *P. notoginseng* to acquire precise full-length transcripts for single-molecule long-read sequencing. In total, three cDNA libraries of different size ranges (1–2 Kb, 2–3 Kb and 3–6 Kb) were constructed using a PacBio RSII sequencing system. These PacBio SMRT Bell libraries were sequenced with 8 SMRT cells, yielding 495,016 reads of inserts (Table 1). A total of 237,368 FLNC reads with complete transcripts from 5′ to 3′ end were obtained based on the inclusion of barcoded primers and the 3′ poly (A) tails.

**Table 1:** Summary of PacBio single-molecular long-read sequencing in *P. notoginseng*.

	1–2 K	2–3 K	3–6 K	Total
Number of reads of insert	147,356	194,966	152,694	495,016
Number of 5′ reads	105,118	133,596	85,856	324,570
Number of 3′ reads	105,938	137,392	83,085	326,415
Number of poly(A) reads	104,939	130,169	72,024	307,132
Number of filtered short reads	8,037	10,880	2,991	21,908
Number of non-full-length reads	8,037	70,788	99,487	178,312
Number of full-length reads	93,284	113,298	50,216	256,798
Number of full-length non-chimeric reads	92,818	112,945	31,605	237,368
Average full-length non-chimeric read length	1739	2,220	3,552	

Messenger RNAs from the five tissues (roots, stems, leaves, flowers, and rhizomes) were sequenced on the Illumina HiSeq 2000 platform to quantify levels of gene/isoform expression and correct single-molecule long-reads. In total, we obtained approximately 310,758,920 100-base pair (bp) paired end (PE) reads, and produced approximately 31 Gbp from these five tissues (Supplementary Table S1). These short reads were employed to further improve contigs and correct small indels and single nucleotide variant (SNV) errors of SMRT-reads by LSC2.0^34^ after removing redundant sequences using cd-hit^35^ (https://www.bioinformatics.org/cd-hit/). A total of 124,589 transcripts sequenced by SMRT were corrected by SGS short reads, and 51,040 non-redundant transcripts (also called as unigenes) were generated as the reference transcriptome of *P. notoginseng* (Supplementary Table S2). Of them, a total of 49,632 transcripts (97%) from PacBio sequencing were successfully annotated based on sequence similarities using BLAST searches against the NCBI non-redundant protein (Nr), non-redundant nucleotide (Nt), Swiss-Prot protein (Swiss-Prot), Kyoto Encyclopedia of Genes and Genomes (KEGG), Cluster of orthologous groups (COG) and GO database (Supplementary Figures S2-6; Supplementary Tables S3–S5).

To validate the length advantage of such a high-quality reference transcriptome, we compared the transcripts independently generated using SGS and SMRT platforms. The SGS short reads of the five different tissues were de novo assembled using Trinity (v2.8.4)^36,37^, yielding a contig N50 of 1.6 Kb and max length of 16,703 bp. In comparison, lengths of SMRT transcripts increased significantly with contig N50 of up to 2.9 Kb and the max length of 20,087 bp (Supplementary Table S2). DETONATE^38^ (https://deweylab.biostat.wisc.edu/detonate/) and Ex90N50 values were employed to assess the completeness of the transcriptome assembly. Our results showed that the score of SMRT (− 23,383,278,787.83) was higher than that of SGS (− 25,093,185,054.93) using DETONATE. SMRT-based transcriptome assembly similarly had larger Ex90N50 value (1984) than SGS-based transcriptome assembly (1965). The obtained results together demonstrate the advantage of SMRT sequencing technology to generate high-quality transcriptome assembly compared to the SGS sequencing platform (Supplementary Tables S7–S8).

**Figure 1:**
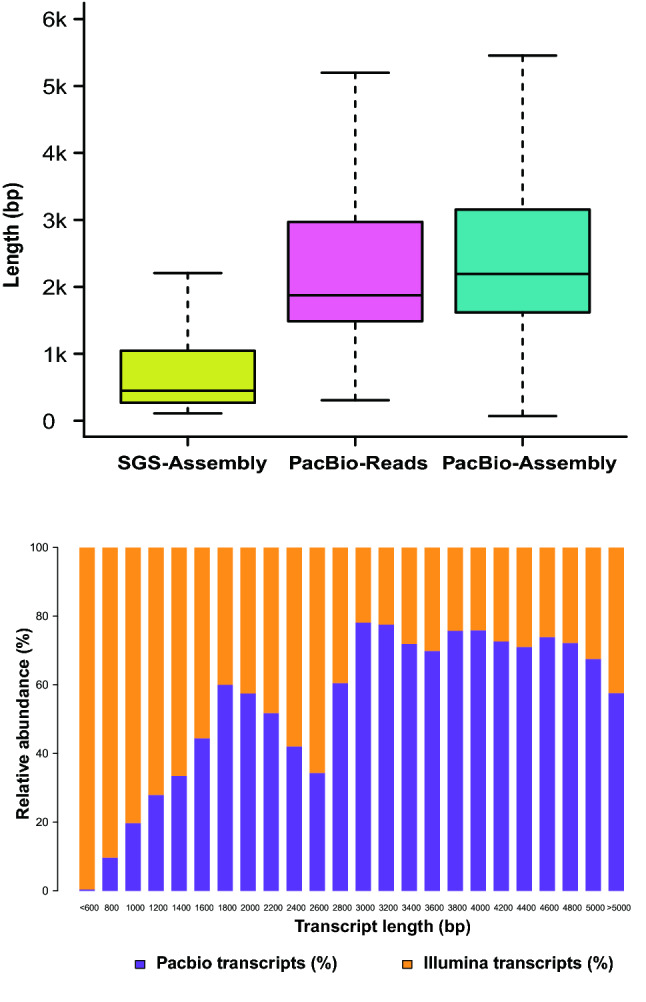
Comparison of transcript length distribution between Illumina and PacBio sequencing platforms. (**A**) Length distribution of SGS transcripts (yellow), SMRT reads (pink), SMRT transcripts after correction (light blue). (**B**) Comparison of transcript length distribution from different sequencing platforms.

The comparative analyses showed that SMRT-reads alone were longer than the assembled transcripts using SGS reads, which become even longer after error correction and transcriptome assembly (Fig. 1A). Most transcripts from SGS were shorter than 1 Kb, whereas the majority of transcripts assembled from PacBio reads ranged from 1.5 to 3 Kb (Fig. 1B). These results demonstrate the advantage of PacBio sequencing technology, through which we obtained high-quality SMRT transcripts corrected by Illumina short reads. Instead of the previous reported SGS-based transcriptomes^18,19^, we first obtained a relatively credible full-length transcriptome of *P. notoginseng* to ensure subsequent data analyses.

### Single-molecule real-time transcript sequencing facilitates the *P. notoginseng* genome annotation

**Figure 2:**
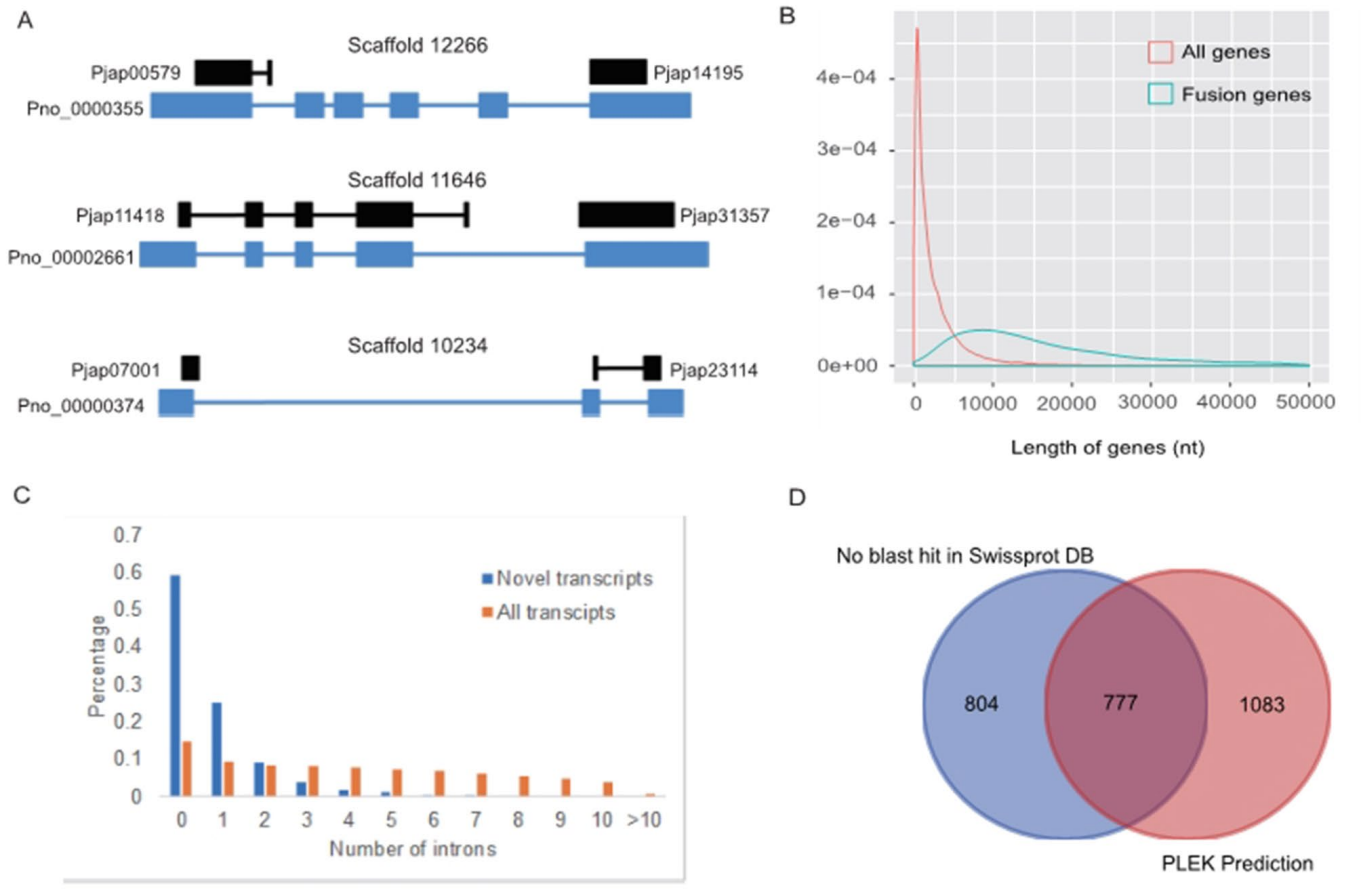
Identification of mis-annotated split genes and unannotated loci. (**A**) Gene structure shown mis-annotated split genes. The black box represents gene loci of genome, the blue box represents exon and solid lines were intron. (**B**) Length distribution of mis-annotated fusion genes and all the genes. (**C**) The intron distribution among novel transcripts and all transcripts. (**D**) Venn diagram showing the intersection of the long non-coding RNA (lncRNA) number from two predicted ways, one is from no BLAST hits in the SWISPROT database, and the other is predicted by PLEK.

In our previously published *P. notoginseng* genome assembly, we predicted 34,369 gene models, with transcript or homology-based evidence for over 27,000 genes^3^. To improve the gene annotation we mapped the FLNC reads to the gene set annotated in the *P. notoginseng* genome based on SGS platform^3^. Our results showed that 2,383 of single contiguous FLNC reads were able to cover the adjacently annotated genes (Fig. 2A). In total, 1,925 genes were mis-annotated as split genes, which could be further merged into up to 927 new genes with the assistance of the FLNC reads (Supplementary Table S9). Comparative analysis showed that these mis-annotated genes were much longer than others, making them difficult to be well-assembled using short reads of SGS technologies (Fig. 2B). We assessed the obtained 8,111 novel transcript isoforms using FLNC reads, yielding 247 isoforms without overlapping the genome assembly, 7,810 isoforms mapped to the intergenic regions and 54 isoforms mapped to the intron regions. Our results suggest that they might come from mis-assembled or mis-annotated loci. Functional annotation of novel isoforms resulted in a total of 71 transcripts from the major gene families involved in the ginsenoside biosynthesis, including 52 *CYP450*, 10 *UGT*, 8 *SE*, and one *SE* (Supplementary Table S10). Furthermore, we found that these novel transcript isoforms displayed fewer introns than other transcripts (Fig. 2C). Among 8,111 novel transcript isoforms, we annotated 2,664 long non-conding RNA (lncRNA) isoforms (Fig. 2D), of which 1,083 isoforms failed to match any entries by SWISSPROT, 804 isoforms were predicted using non-coding RNA prediction software, PLEK (Version: 1.2)^39^, and 777 isoforms were predicted by the two above-mentioned methods (Fig. 2D). Our analysis thus proves the power of the SMRT sequencing technology to identify novel genes/isoforms and correct incompletely assembled loci in *P. notoginseng*. Similar efforts have been put to the gene annotations of *Sorghum bicolor*^26^, *Phyllostachys edulis*^27^, and switchgrass^32^, which were significantly improved based on FLNC reads, highlighting the potential of single-molecule long-read sequencing for the genome annotation.

### The full-length transcriptome has efficiently assisted a widespread identification of AS events

**Figure 3:**
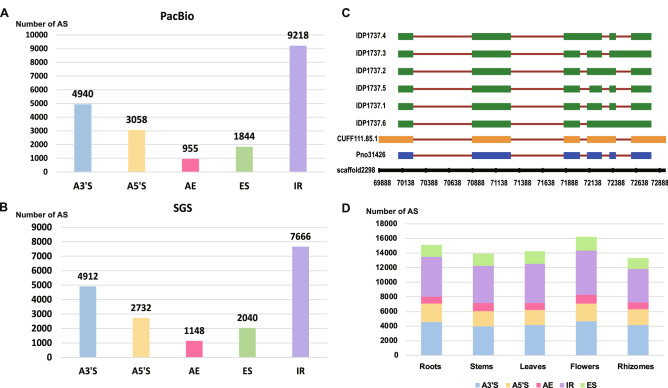
Alternative splicing events of *P. notoginseng*. (**A**) The different alternative spliced types in SGS transcriptome of *P. notoginseng*; (**B**) The different alternative spliced types in PacBio sequencing data of *P. notoginseng*; (**C**) An exemplar gene (Pno31426) produces seven splice isoforms. Gene model (blue), splice isoforms with SMRT reads (green), splice isoforms with Illumina reads (orange); (**D**) Distribution of different types of alternative splicing events in genes involved in the ginsenoside biosynthesis pathway from five tissues of *P. notoginseng*. S01 is root, S02 is stem, S03 is leaf, S04 is flower, and S10 is rhizome. ES, exon skipping; IR, intron retention; A3′S, alternative 3′splice site; A5′S, alternative 5′ splice site.

It has been proven that long reads generated by SMRT sequencing platform are suitable to extensively and accurately identify alternative splicing (AS) forms^40,41^. The SGS was formerly employed to identify novel introns and splicing variants in plants, such as *Oryza sativa*^42^, *Arabidopsis thaliana*^43,44^, *Brachypodium distachyon*^45^ and *Glycine max*^24^, showing that almost 60% of multi-exon genes occurred AS events^46^. In this study, we first identified AS events from the Illumina short reads using SUPPA^47^ after aligning to the *P. notoginseng* genome through Tophat^48^. We detected junctions in 43,775 Illumina transcripts associated with 9,550 genes (Supplementary Tables 11–12). A total of 18,498 AS events identified in the Illumina transcriptome of *P. notoginseng* were further classified into the five distinct types: 2,732 alternative 5′donor (15%), 4,912 alternative 3′ donor (27%), 1,148 alternative exon (6%), 7,666 intron retention (41%), and 2,040 exon skipping (11%) events (Supplementary Tables S11,S12; Fig. 3A). Meanwhile, we detected junctions in 21,981 transcripts generated from long SMRT reads, which were associated with 6,324 genes, through isoform detection and prediction using IDP^49^ and SUPPA^47^, respectively, after aligning to the *P. notoginseng* genome assembly^3^ using GMAP^50^. From spliced alignments of the long-read SMRT sequences, IDP and SUPPA analyses yielded 20,015 isoforms. They were further divided into the five types: 3,058 alternative 5′ donor (15%), 4,940 alternative 3′ donor (25%), 955 alternative exon (5%), 9,218 intron retention (46%), and 1,844 exon skipping events (9%) (Supplementary Table S11; Supplementary Table S13; Fig. 3B). Our results showed that intron retention comprised the majority of AS events, which is in good agreement with results observed in *Sorghum bicolor*^26^, *Z. mays*^27^, *Fragaria vesca*^29^, and *Phyllostachys edulis*^30^, *Populus*^31^ and switchgrass^32^. In *P. notoginseng*, more AS events were identified from long SMRT than short Illumina reads, whereas genes occurred alternative-splicing forms from the short Illumina reads exceeded long SMRT reads. For example, we detected seven AS isoforms in the exemplar gene (*Pno31426*); of them, only one could be found using the Illumina short reads while six were identified in the SMRT long reads (Fig. 3C). It is well recognized that SGS is limited to assembling full-length transcripts for the AS detection due to short reads and PCR amplification bias during library construction^51^. The SMRT sequencing technology, however, is able to overcome these defects to have proven the advantage to detect AS events in many other plant species, such as *Salvia miltiorrhiza*^25^*,* moso bamboo^30^, strawberry^29^, maize^27^ and sorghum^26^. Considering the observation that AS may occur in a highly tissue-specific manner^27^, we compared differential splicing events detected in roots, stems, leaves, flowers and rhizomes of *P. notoginseng*. Among the five tissues, flowers harbored the largest number (16,224; 22.28%) of splicing isoforms, followed by roots (15,084; 20.72%), leaves (14,246; 19.57%) and stems (13,903; 19.10%), whereas rhizomes had the fewest number (13,342; 18.33%) (Fig. 3D; Supplementary Table S14). Our results are in good agreement with findings of tissue-specific isoforms and alternative splicing modes in maize, which showed that pollen had the highest proportion of tissue-specific isoforms (9,842; 61.3%), whereas root had the lowest (13,386; 44.6%)^27^. The abundance of tissue-specific isoforms in flowers may associate with the specialized function of reproductive activities, for example, the pollination in *P. notoginseng*.

### Genome evolution of the *Panax* species

**Figure 4:**
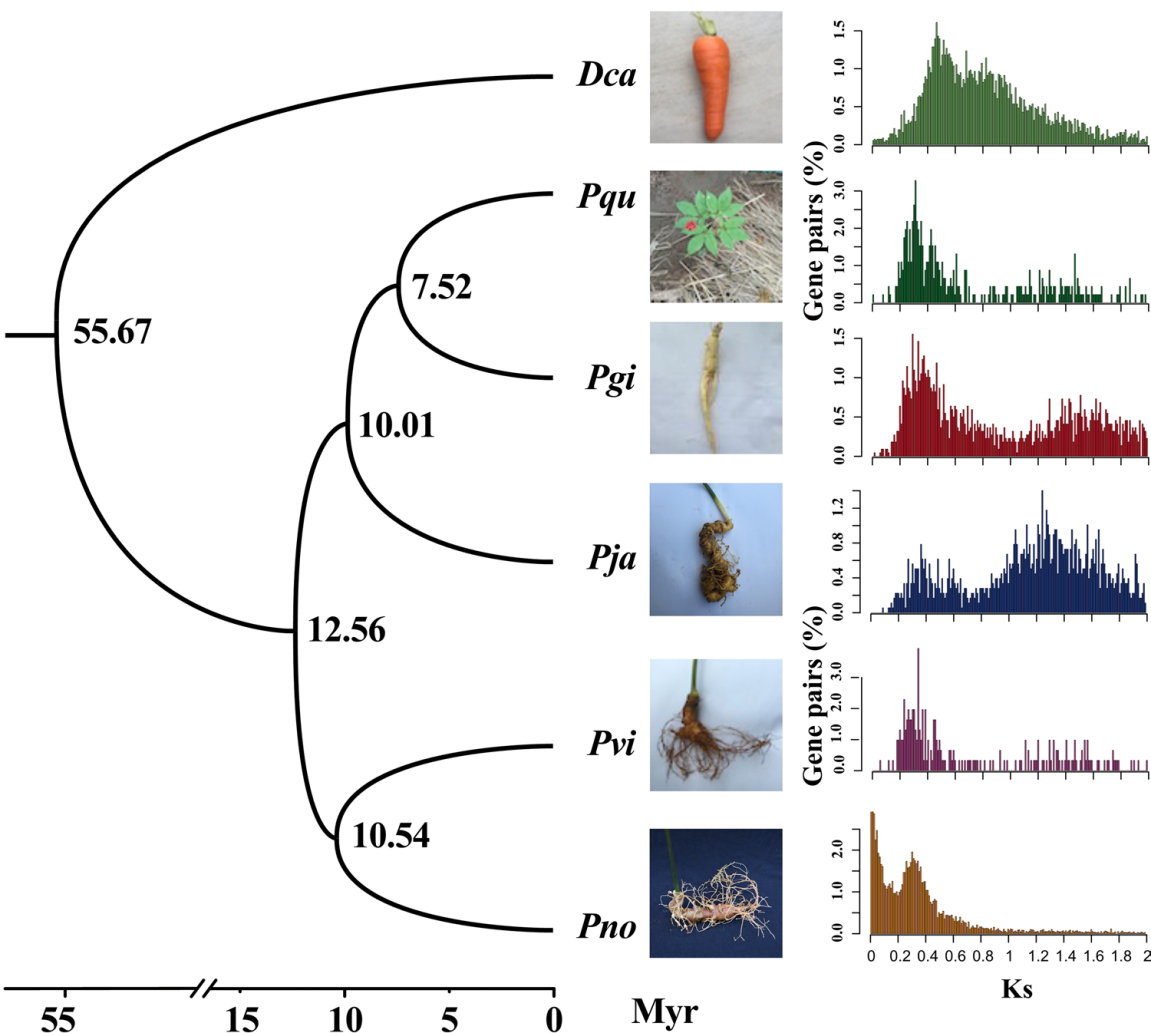
The WGD events detected among the five *Panax* species based on the transcriptome datasets. Species names were abbreviated as follows: Pno, *P. notoginseng*; Pgi, *P. ginseng*; Pqu, *P. quinquefolium*; *Pja, P. japonicas*; Pvi, *P. vietnamensis*; Dca, *D. carota.* The Ks values between each gene pairs were estimated based on the NG (Nei & Gojoberi) method of Yang that implemented in the PAML package.

The acquisition of the high-quality reference transcriptome by SMRT sequencing technology permits us to broaden our knowledge about the genome evolution of the *Panax* species. We analyzed and compared the five representative *Panax* species, including *P. notoginseng*, *P. ginseng*, *P. quinquefolium, P. japonicas* and *P. vietnamensis*, based on their high-quality transcriptome sequences^52–55^ (Supplementary Fig. 7; Supplementary Tables S15,S16). Using the 1,416 single-copy orthologous gene families identified using OrthoMCL^56,57^, we reconstructed their phylogenetic relationships using the carrot as outgroup by RAxML package (version 8.1.13)^58,59^. The obtained phylogenetic trees were visualized using MEGA (version 6)^60–63^ (Fig. 4; Supplementary Figs. S8–9; Supplementary Table S17). Results showed that the two diploid species, *P. notoginseng* and *P. vietnamensis*, grouped together with a strong bootstrap support, while the three other tetraploid species, including *P. ginseng*, *P. quinquefolium, P. japonicas*, formed the other cluster with sufficient bootstrap supports.

Previous studies on the sequenced plant genomes have shown that polyploidy has been a prominent feature in the evolutionary history of angiosperms and that whole-genome duplication (WGD) events, in particular, have had major impacts on crop gene and genome evolution^64–68^. Our genome analysis detected that one round of a whole genome duplication (WGD) event has occurred ~ 26.15 Million Years Ago (MYA) in *P. notoginseng*^3^. To detect the occurrence of this WGD event in *Panax* species, we identified 2,769 paralogous gene pairs, based on the *Ks* distribution of paralogous gene pairs (Supplementary Fig. S10). On the basis of these duplicated gene pairs, we calculated an age distribution of synonymous substitution rates (*Ks*) that peaked around ~ 0.34 in *P. notoginseng*, *P. ginseng*, *P. quinquefolium, P. japonicas*, and *P. vietnamensis* (Fig. 4; Supplementary Fig. S10). Our results demonstrate that this WGD event occurred in the common ancestor of the studied *Panax* species, which is strongly supported by the tetraploid *P. ginseng* genome analysis that revealed such a shared WGD event in the *Panax* lineage at 28 MYA (Pg-β)^69^.

### Comparative metabolic and transcriptomic analyses reveal the ginsenoside biosynthetic machinery

**Figure 5:**
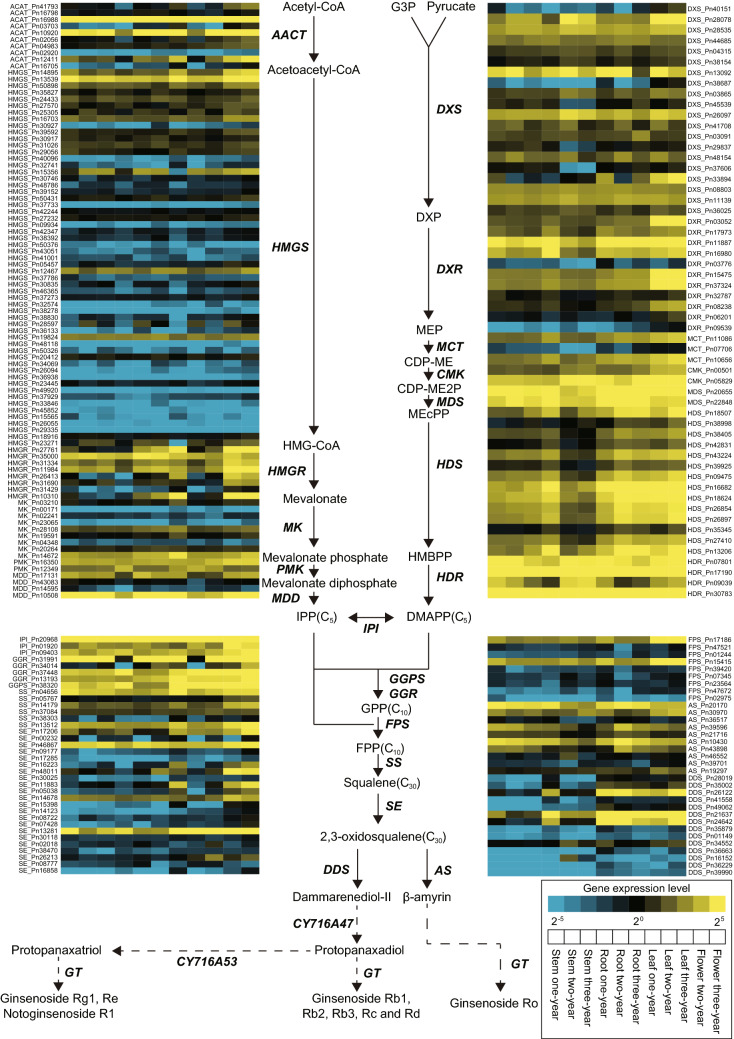
A heat map of differentially expressed genes involved in the ginsenoside biosynthesis among the eleven developmental tissues collected from the 1-year old, 2-year old and 3-year old plants of *P. notoginseng*. The heat map was drawn based on the mean TPM of the three biological repeats, of which high to low expression levels were indicated from yellow to blue in color; 11 boxes represent different tissues collected from the 1-year old, 2-year old and 3-year old plants of *P. notoginseng*.

Ginsenosides function as the major pharmacologically active compounds of *P. notoginseng*, of which more than 60 have been characterized from this plant^9^. The availability of high-quality SMRT transcriptome of *P. notoginseng* allows us to better know the ginsenoside biosynthetic machinery. Here we obtained the full-cDNA sequences of twenty-three gene families involved in the cytosolic mevalonic acid (MVA-) and plastidial 1-deoxy-d-xylulose-5-phosphate (MEP-) pathways (Supplementary Table S18). These gene families included *ACAT, AS, CMK, DDS, DXR, DXS, FPS, GGPS/GGR, GPS, HDR, HDS, HMGR, IPI, HMGS, MCT, MDD, MDS, MK, PMK, SE*, *SS*, *CYP450* and *GT.* We characterized a total of 215 unigenes involved in the terpenoid backbone biosynthesis, of which *CYP450* and *GT* were the two largest gene families with 170 and 189 unigene isoforms, respectively. To examine expression patterns of the above-mentioned genes among different tissues in *P. notoginseng*, we produced RNA-Seq datasets of 33 RNA samples from 11 tissue/age combinations of the one-year, two-year and three-year old plants (Supplementary Fig. S11). To ensure the reliability of tissue-specific expression profiling of these genes we collected RNA-Seq datasets from three biological replicates for each of these tissues (Supplementary Table S19). The Pearson Correlation Coefficient (PCC) was employed to calculate the correlation of gene expression levels (Fragments Per Kilobase per Million mapped fragments (FPKM) using RSEM v1.3.1^70^) among the three repeated samples of the 11 tissue/age combinations from the 1-year, 2-year and 3-year old plants of *P. notoginseng* (Supplementary Fig. 12). We calculated expression levels of genes involved in the ginsenosides biosynthesis in different tissues. We used these unigenes obtained from the SMRT transcriptome as a reference, and individually mapped RNA-Seq reads from the five tissues of three-year old plants to them (Supplementary Table S20). The expression levels for each gene were calculated with Transcripts Per Million (TPM) using Salmon^71^ (Supplementary Table S21), which were then visualized by the heat map. Considering that post-transcriptional alternative splicing isoforms existed for most genes, we selected the longest transcripts with the highest levels of gene expression to present tissue-specific expression profiling (Supplementary Fig. S13). Tissue-specific expression profiling of these eleven tissues from different developmental stages revealed that these twenty-one gene families involved in the terpenoid backbone biosynthesis were differentially expressed. Gene expression profiling using the longest transcript for each gene family as a representative showed that at least eleven gene families, including *ACAT, CMK, DDS, DXS, DXR, GGR_GGPS, HMGR, MDD, MDS, PMK* and *SE*, were most highly expressed in the flowers (Supplementary Fig. S13A). In addition, the five gene families (*HDS, MDS, GGPS_GGR, DDS* and *AS*) were highly expressed in leaves, but only two (*HDR* and *CMK*) were highly expressed in roots (Supplementary Fig. S13A). Furthermore, the top expression profiling showed that the majority of 17 gene families (*DXS, DXR, MCT, CMK, MDS, HDS, ACAT, HMGS, HMGR, PMK, MDD, GGPS_GGR, SS, SE, DDS, MDD* and *IPI*) were highly expressed in flowers, and seven gene families (*MCT, MDS, HDS, IPI, GGPS_GGR, DDS* and *AS*) were highly expressed in leaves, but only four (*DXS*, *CMK*, *MDD* and *ACAT*) were highly expressed in roots (Supplementary Fig. S13B). Similar patterns were observed while incorporating all three biological replicates into data analyses of differentially expressed genes involved in the ginsenoside biosynthesis among the eleven developmental tissues collected from the one-year old, two-year old and three-year old plants of *P. notoginseng* (Fig. 5; Supplementary Fig. S14; Supplementary Tables S21–22). Our results altogether indicate that the ginsenoside biosynthesis of *P. notoginseng* may predominantly occur in flowers, followed by leaves and roots.

Based on previous studies on the biosynthesis of ginsenosides^11,72^, we obtained the twenty-three gene families involved in the ginsenoside biosynthesis of *P. notoginseng* and their orthologs in the other four *Panax* species, including *Panax ginseng*^52^, *Panax quinquefolium*^53^, *Panax japonicas*^55^, *Panax vietnamensis*^54^, and *Daucus carota*^73^ (Supplementary Table S23). Phylogenetic analyses showed that *P. notoginseng* has experienced lineage-specific amplification in thirteen gene families compared with the three other *Panax* species, including *ACAT*, *AS*, *DDS*, *DXR*, *DXS*, *HDS*, *HMGS*, *MDD*, *MK*, *SE*, *SS, UGT* and *CYP450* (Supplementary Fig. S15). The functional divergence of these duplicated genes, known as neofunctionalization, may enhance the diversification of the specified ginsenoside biosynthesis of *P. notoginseng*.

To provide insights into the biosynthesis and accumulation of ginsenosides in *P. notoginseng* we performed high-performance liquid chromatography analysis (HPLC) of 11 tissue/age combinations representing different developmental stages from one-year, two-year and three-year old plants of *P. notoginseng*. We totally detected 17 characteristic metabolites (Rb1, Rb2, Rd, Re, R1, Rg1, Rh1-1, Rh1-2, F1, F2, Rg3-1, Rg3-2, CK, Rh2, Fa, Fc and Fe) (Supplementary Table S24), which quantitatively and qualitatively varied among them. For each tissue, we identified diverse types of ginsenosides, of which one-year old plants obviously had fewer phytochemical types of characteristic secondary metabolites than two-year and three-year old plants. Of these 17 characteristic secondary metabolites, one-year old roots only contained 9 types of ginsenosides, stems had 6 types, and leaves had 11 types; two-year old plant had 13, 6, 13 and 7 types in roots, stems, leaves and flowers, respectively; in three-year old plants, there were 13, 8, 11 and 9 in roots, stems, leaves and flowers, respectively (Supplementary Table S25). It is apparent that total contents of different types of ginsenosides gradually accumulated with the growth of the *P. notoginseng* plants. Among different tissues of *P. notoginseng*, roots interestingly appeared the most variable in terms of contents and types of ginsenosides and thus act as active compounds; ginsenosides are extraordinarily abundant in roots of the three-year old plants. Similar to previous results^74^, we found that roots were rich in protopanaxatriol- and protopanaxadiol-type saponins, whereas protopanaxadiol-type saponins predominated in aerial parts, including leaves, stems and flowers.

We further investigated the AS events of genes involved in the ginsenoside biosynthesis of *P. notoginseng*. Our results showed that the AS events are quantitatively specific in the twenty-one ginsenoside biosynthesis genes; especially, *HMGR* from MVA pathway exhibited ten AS isoforms, *DXS* from MEP pathway had eleven AS isoforms, and the GGPS_GGR and SE from cyclizing had ten and nine AS isoforms, respectively (Supplementary Fig. S16). These findings indicate that the regulation of alternative splicing might play an important role in controlling flux through both the MEP- and MVA-dependent pathways to achieve the ginsenoside biosynthesis.

## Conclusions

This is the first study based on PacBio-based transcriptomic data in the genus *Panax*, to the best of our knowledge. We employed Pac-Bio SMRT and Illumina SGS sequencing technologies for a variety of tissues, including roots, rhizomes, stems, leaves and flowers, to provide a more widespread transcriptome atlas of *P. notoginseng*, a famous traditional Chinese medicine. The obtained results have established a rich data set of FL cDNA sequences and largely extended attainable transcriptomic data resources through a genome-wide identification of novel alternative splicing events in *P. notoginseng*. We applied single-molecule long-read sequencing to producing full-length non-chimeric (FLNC) reads, demonstrating the PacBio Iso-Seq platform’s powerful utility in identifying novel genes/isoforms and particularly correcting poorly assembled loci in the previously reported SGS-based genome assembly. We identified more AS events from long SMRT reads than short Illumina reads, including a large number of AS events associated with the MEP- and MVA-dependent pathways of the ginsenoside biosynthesis. The completion of full-length transcriptome sequences of *P. notoginseng* has greatly enhanced our understanding about genome evolution of the *Panax* species, confirming a shared WGD event in the *Panax* lineage with *Ks* peaked around ~ 0.34. The availability of high-quality SMRT transcriptome of *P. notoginseng* also permits us to obtain the full-cDNA sequences of twenty-three gene families involved in the MVA- and MEP- pathways of the ginsenoside biosynthesis. On the basis of an almost full-length reference transcriptome as well as the comprehensive transcriptome and metabolic landscape of *P. notoginseng* we better identified specific genes and obtained in-depth insights into metabolic pathways that will enable to elucidate the accumulation and mechanisms underlying the ginsenoside biosynthesis in *P. notoginseng*. Together, this study provides rich transcriptomic and metabolic datasets, which should help uncover the ginsenoside biosynthetic machinery and lay a solid foundation for future genetic breeding program and empirical metabolic engineering in *P. notoginseng*.

## Materials and methods

### Plant materials and RNA extraction

Three-year old *P. notoginseng* plants were harvested in Wenshan County, Yunnan Province, China. The root, stem, leaf, flower and rhizome tissues were collected in August, 2015. These tissues were cut into small pieces and stored at -80℃ after being quickly frozen in liquid nitrogen. Meanwhile, the other 33 *P. notoginseng* samples were collected, including three tissues (root, stem and leaf) from one-year old plant, four tissues (root, stem, leaf and flower) from two-year old plant and three-year old plant, respectively. We took the three individual plants at a similar development stage under the same habitat, in which different tissues were sampled to set the three biological replicates. Note that only three tissues were collected from one-year old plants, as they cannot develop flowers throughout a year. Total RNA was extracted using the TRIzol (TRIzol Reagent Invitrogen, Beijing, China, No. 15596-026) processed following the protocol provided by the manufacturer. The RNA integrity was assessed with an Agilent 2,200 TapeStation (Agilent Technologies, https://www.agilent.com/).

### cDNA library preparation and Illumina sequencing

The poly (A) + RNA (mRNA) was respectively isolated from the total RNA pool using Dynal oligo (dT) 25 magnetic beads according to the manufacturer’s instructions. After purification, the fragmentation buffer (Ambion) was used to fragment the mRNA into smaller pieces. Then, the SuperScript III reverse transcriptase and N6 random hexamers were used to synthesize the first-strand cDNA of the cleaved RNA fragments, and RNase H and DNA polymerase were used to synthesize the second strand cDNA. HiSeq libraries were prepared using the Illumina TruSeq Stranded mRNA kit. Subsequently, these cDNA fragments were processed by an end repair and the ligation of adapters followed the manufacturer’s protocol. The products were further purified and enriched with PCR for preparing the final sequencing cDNA library. The library quality was detected by Agilent 2100 Bioanalyzer. The cDNA library was sequenced from both 5′and 3′ends using the Illumina HiSeq 2000 platform by following the manufacturer’s instructions protocol.

First, the five tissues including root, stem, leaf, flower and rhizome of three-year old *P. notoginseng* were sequenced on Illumina HiSeq 2000 platform, each of which generated more than 4 Gb paired-end (PE) reads data with read length of 100 bp. They were used to correct the above sequenced SMRT reads and identify alternative splicing isoforms. These data were assembly by Trinity (v2.8.4) ^36,37^ with parameter: Trinity.pl –seqType fq –max_memory 600G–left reads_1.fq–right reads_2.fq–CPU 6 –output trinity_out–full_cleanup–min_contig_length 250. The cd-hit (v4.6.8) was then used to remove redundant sequences. To assess the completeness of the transcriptome assembly we employed Ex90N50 and DETONATE^38^ (parameter: first step: rsem-eval-estimate-transcript-length-distribution S_Trinity_clean.fa length_distribution_parameter.txt. second step: rsem-eval-calculate-score –p 8 –transcript-length-parameters length_distribution_parameter.txt –paired-end –phred33 –strand-specific left.fastq right.fastq S_Trinity_clean.fa assembly1_rsem_eval 300). On the other hand, the 33 RNA sample from different tissues of different development stage (one-year old: root, stem and leaf; two-year old and three-year old: root, stem, leaf and flower) were sequenced by Illumina HiSeq 2000 platform.

### Library preparation and SMRT sequencing

Total RNAs (10 μg) with an RNA integrity number (RIN) values larger than 8.5 were equally mixed from the five different tissues including rhizome, root, stem, leaf and flower. Next, the cDNA synthesis was finished by The Clontech SMARTer PCR cDNA Synthesis Kit (cat. No.634925, http:/www.clontech.com/), where the reverse transcription (RT) was primed with an anchored oligo (dT) 30 primer.

The product was separated by a BluePippin size selection system (Sage Science, https://www.sagescience.com/) into 1–2 kb, 2–3 kb and 3–6 kb. The three SMRT bell libraries were constructed using 500 ng size-selected cDNA with Pacific Biosciences SMRT bell TEMPLATE Prep kit 1.0 (part 100- 259–100, https://www.pacb.com/) according to the standard protocol. The DNA/Polymerase Binding Kit P5 and v2 primers were used to conduct binding of SMRT bell templates to polymerases. The libraries were subsequently sequenced on the PacBio RS II real-time (RT) sequencer platform by C3 reagents with 120 min movies, with a total of eight SMRT cells, in which the 1–2- and 2–3-kb libraries were sequenced using three SMRT cells, respectively, while the 3–6-kb library used two SMRT cell.

### Data analysis of *PacBio* SMRT long-reads

Using RS_IsoSeq (2.3v) to analyze PaciBio single-molecule long reads, the smrtanalysis_2.3.0.140936.p4.150482 was used from the command line to obtain insert reads. Next, the script pbtranscript.py of smrtanalysis_2.3.0140936.p4.150482 was used for the characterization of the full-length reads. The Clontech kit was used to identify the 5′ and 3′ primers, and the poly (A) tail before 3′ primer is an important signal to discriminate strand-specific full-length reads. The LSC 2.0 (https://www.healthcare.uiowa.edu/labs/au/LSC/) (parameters: LSC-2.0/bin/runLSC.py –long_reads SQ_SMRT.fa –short_reads SQ_Illumina.fa –output output) was used to correct the sequencing errors in consensus reads using Illumina reads from the five different tissues of *P. notoginseng*. DETONATE^38^ and Ex90N50 value were again employed to assess the completeness of SMRT-based transcriptome assembly as above described for the quality evaluation of Illumina-based transcriptome assemblies.

### Gene expression analysis

To assess levels of gene expression, RSEM^70^ (v1.3.1) was used to map RNA-seq reads from the five tissues of three-year old *P. notoginseng* plants to the SMRT-based reference transcriptome and calculate FPKM value. The parameters were adopted as below: the first step, rsem-eval-estimate-transcript-length-distribution S_Trinity_clean.fa length_distribution_parameter.txt; and the second step: rsem-calculate-expression–paired-end-no-bam-output–alignments -p 8 input_Aligned.toTranscriptome.out.bam reference_name out_prefix. The results were visualized and clustered using R package. The correlation of gene expression levels among the three repeated samples of the 11 tissue/age combinations from the 1-year old, 2-year old and 3-year old plants of *P. notoginseng* were calculated through the Pearson Correlation Coefficient (PCC) by R package (version 3.0.1). edgeR was further employed to add statistical rigor to our analyses of all biological replicates related to gene families involved in the ginsenoiside biosynthesis pathway. In addition, we used another method to calculate gene expression levels, GSNAP (version 2017-12)^75^ were used to reads map with default parameters, and gene expression levels were evaluated by Transcripts Per Million (TPM) using Salmon (0.11.0)^71^.

### Transcript isoform mapping and novel isoform prediction

The corrected SMRT sequences were aligned against the *P. notoginseng* reference genome using GMAP^50^ aligner v2016-08–24 with the parameters: –min-identity 0.95 and –allow-close-indels 2. We identified the novel isoforms according to the following three criteria. We identified an isoform as novel if it met any of the following three criteria: (1) the isoforms cannot map to the reference genome of *P. notoginseng*; (2) the isoforms can map to introns of genes; (3) the isoforms can map to intergenic regions. Then, we employed the Blastx (version 2.2.26) to align the novel isoforms against SWISS-port database, which were then classified into can protein-coding isoforms and non- protein -coding isoforms. The protein-coding isoforms were further used to perform GO enrichment and PFAM analysis by BLAST2GO^76^ with default parameters. On the other hand, the novel transcript isoforms were used to search for the homologs against the plant lncRNA database, GreeNC and CANTATdb, using blast 2.6.0 + with a threshold E-value of 10^–5^. lncRNA was predicted by PLEK^39^ (https://sourceforge.net/projects/plek/files/). Note that we used a different PLEK model trained on the other plant transcriptome data including rice and tea tree before *P. notoginseng* in this study.

### Identification of AS events

In order to categorize the alternative splicing events, SMRT reads were mapped to the *P. notoginseng* genome by GMAP^50^ to identify alternative splicing isoforms. Based on the above mapped GTF format files, the software SUPPA^47^ (parameter: python ~ /SUPPA/suppa.py generateEvents -f ioi -i ~ /reference.formatted.gtf -o ./out.isoforms) and IDP^49^ (parameter: python runIDP.py run.cfg 0) were employed to detect AS isoforms. In addition, after the Illumina RNA-seq reads were assembled by Cufflinks (version 2.1.1)^77^.Tophat (version 2.1.0)^48^ was used with parameters (tophat -N 5 –read-edit-dist 5 -r 50 –mate-std-dev 20 -p 20 -a 10 -i 20 -o filter_out_T15/ filter S61-T15_S61-T15-I_good_1.fq S61-T15_S61-T15-I_good_2.fq) to align to the *P. notoginseng* genome.

### *Ks* calculation and identification of whole-genome duplication events

We first identified the paralogous gene pairs using a combination of OrthoMCL^56,57^ and Blast-based methods, yielding a total of 2,769 paralogous genes pairs in the *Panax* species, including *P. notoginseng*, *P. ginseng*, *P. quinquefolium, P. japonicas* and *P. vietnamensis*, respectively, based on their high-quality transcriptome sequences. We then calculated the number of synonymous substitutions per synonymous site (*Ks*) for these gene pairs based on the NG (Nei & Gojoberi) method implemented in the PAML program (version 4.9b)^78,79^. Finally, the *Ks* distribution for each species was plotted and displayed using R language (version 3.0.1). To estimate the divergence time of *Panax* species, we calculated the *Ks* values of the 1,416 single-copy orthologous gene pairs determined by OrthoMCL. The peak *Ks* value was then converted to the divergence time using the universal substitution rate of 6.5 × 10^–9^ mutations per site per year.

### Phylogenetic analyses

The OrthoMCL^56,57^ package (version 2.0.9) were used to identify the 23 gene families involved in the ginsenoside biosynthesis between and nine other plant species, including grape, kiwifruit, carrot, coffee, pepper, potato, tomato, cacao and rice^3^. The same method was used to identify the 20 related to photoperiod regulate flowering time gene families between the five *Panax* species including *P. notoginseng*, *P. ginseng*, *P. quinquefolium, P. japonicas*, *P. vietnamensis* and *D. carota*. To construct the phylogenetic relationships of protein-coding gene sequences we individually retrieve and align them between *P.notoginseng* and other plant species using MUSCLE (version 3.8.31)^80,81^ with parameter: muscle -in input.fasta -out output.aln. The alignments were further concatenated to construct a super gene tree for each plant species. To determine the best suitable substitution model for the phylogeny reconstruction, we employed the program of ModelTest (version 2.1.7)^82,83^ with default parameters. Results showed that GTR + GAMMA was the best one among nearly 80 tested models. Based on this model, we finally constructed the phylogenetic tree between *P. notoginseng* and the four other plant species using RAxML package (version 8.1.13)^58,59^ using carrot as outgroup. Bootstrap support values were calculated from 1,000 iterations. The obtained trees were visualized using MEGA (version 6.0)^60–63^.

### Ginsenoside extraction and HPLC analysis

The Agilent 1,100 HPLC system equipped with Agilent ZORBAX SB-C18 (4.6*250 mm, 5 μm) was used to measure the ginsenoside (Rb1, Rb2, Rd, Re, R1, Rh1-1, Rh1-2, F1, F2, Rg3-1, Rg3-2, CK, Rh2, Fa, Fc and Fe) (Supplementary Table S25) contents in the 11 tissue/age combinations (including one-year old root, stem and leaf; two-year old root, stem, leaf and flower; and three-year old root, stem, leaf and flower) of *P. notoginseng.* Approximately 1.00 g of dried materials was accurately qualified and powdered, and 70% methanol was used to extract ginsenoside contents and take 10μL of the sample volume to HPLC analysis. The mobile phase was selected as 0.5% (v) formic acid (A) in water, and acetonitrile (B), and the gradient of 8% B for 5 min, 25% B for 23 min and 8% for 25 min were used with a flow rate is 1.0 mL/min. The detection wavelength was set to 280 nm. The target chromatographic peaks were identified by comparing the retention time with their standards. Quantification was calculated by peak integration using the external standard method.

### Accession numbers

These sequence data have been submitted to National Genomics Data Centre under accession number PRJCA002506. Addresses are as follows: https://bigd.big.ac.cn/.

## Supplementary information

Supplementary information.

Supplementary table 9.

Supplementary table 10.

Supplementary table 11.

Supplementary table 12.

Supplementary table 21.

Supplementary table 22.

Supplementary table 25.
