# Supplementary material for: SMRT- and Illumina-based RNA-seq analyses unveil the ginsinoside biosynthesis and transcriptomic complexity in Panax notoginseng
Source: Sci Rep. 2020 Sep 17;10:15310. doi: 10.1038/s41598-020-72291-1 (PMC7499265; doi:10.1038/s41598-020-72291-1)
Supplement: Supplementary file 1 — Supplementary information. [file 41598_2020_72291_MOESM1_ESM.docx]

**Supporting Information Appendix**

**SMRT- and Illumina-based RNA-seq analyses unveil the ginsinoside biosynthesis and transcriptomic complexity in *Panax notoginseng***

**Dan Zhang ^1^*****, Wei Li** ^1^*, **Zhong-jian Chen**^2^**, Fu-gang Wei**^3^**, Yun-long Liu**^4^**, and Li-zhi Gao^1, 4^**^§^

^1^ Institution of Genomics and Bioinformatics, South China Agricultural University, Guangzhou 510642, China

^2^ Wenshan Sanqi Institute of Science and Technology, Wenshan University, Wenshan 663000, China

^3^ Wenshan Miaoxiang Notoginseng Industral Co., LTD, Wenshan 663000, China

^4^ Plant Germplasm and Genomics Center, Kunming Institute of Botany, the Chinese Academy of Sciences, Kunming 650204, China

***Authors who make equal contributions;**

^§^ **Corresponding Author:**

Li-zhi Gao

Tel/Fax: (+0086871) -65223277

E-mail: Lgaogenomics@163.com

**Running title:**

Long-read transcriptome of *Panax notoginseng*

**Supplementary Figures**

**SMRT sequencing of**

***P. notoginseng***

**Illumina sequencing of**

***P. notoginseng***

**Reads**

**mapping**

**Reads**

**mapping**

**Assembly**

**annotation**

**Correction**

***P. notoginseng***

***Genome***

**HPLC analysis**

**The correlation between gene expression and ginsenoside content**

**Ginsenoside biosynthesis pathway analysis**

**Gene expression analysis**

**One, two, three-years old *P. notoginseng* 33 tissues (include 3 repeat) Illumina sequencing**

**High quality reference transcriptome**

**AS identified**

**Ginsenoside biosynthesis pathway analysis**

**Gene expression analysis**

**AS identified, improve annotation of genome, new genes, LncRNA**

**Illumina transcriptome**

**Supplementary Figure 1: Experimental design diagram of this study.**


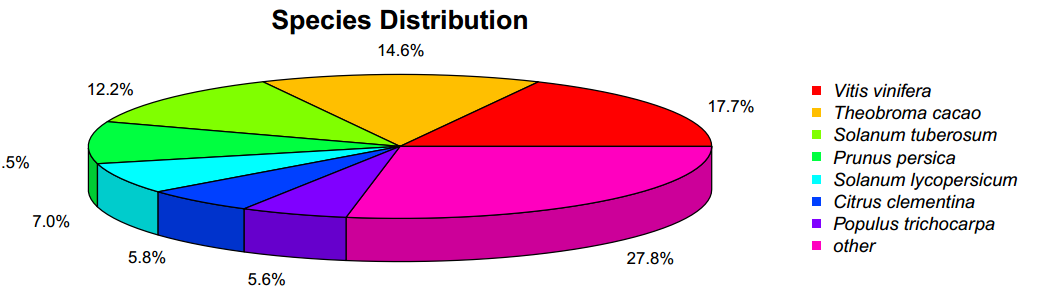


**Supplementary Figure 2**: **The species statistic of transcript blast to Nr database.**


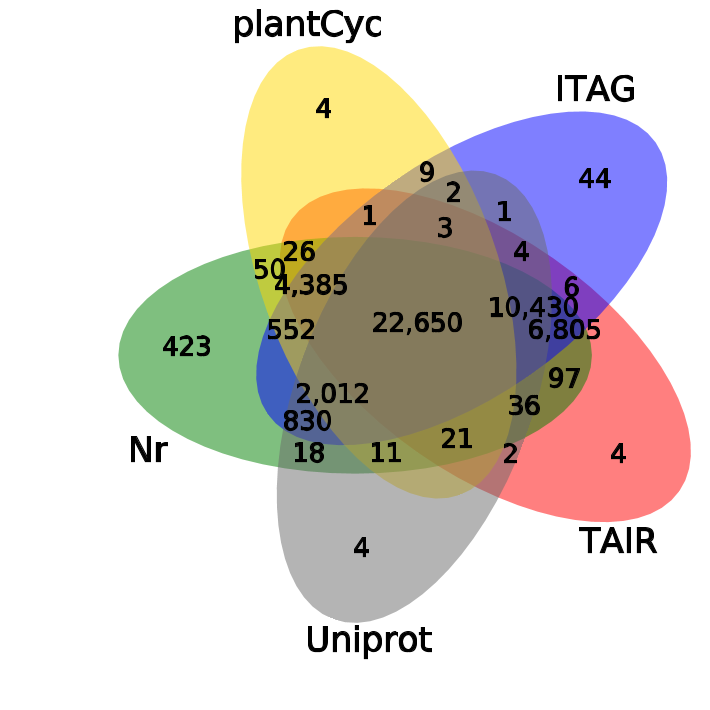


**Supplementary Figure 3: Venn diagram of unigenes from PacBio sequencing annotated using various databases.**


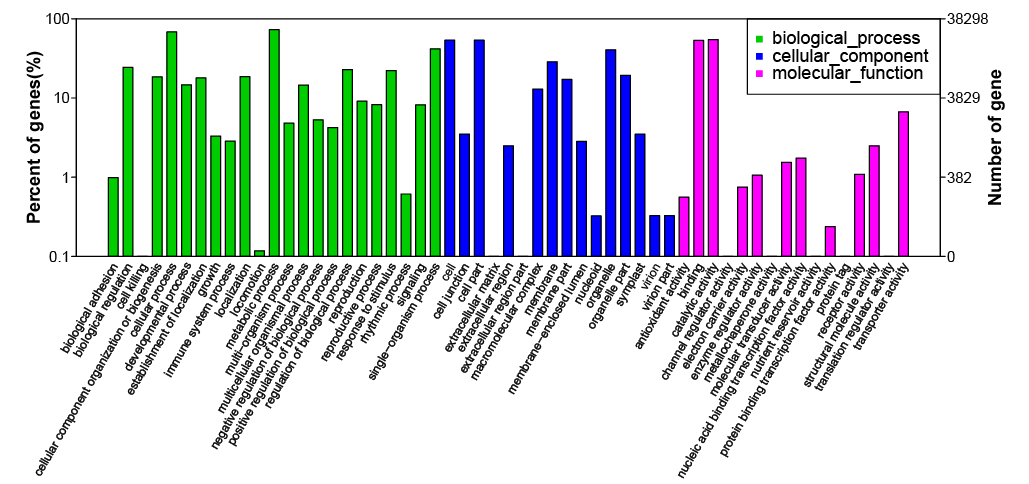


**Supplementary Figure 4**: **Gene Ontology classification of the unigenes of *P. notoginseng*.**


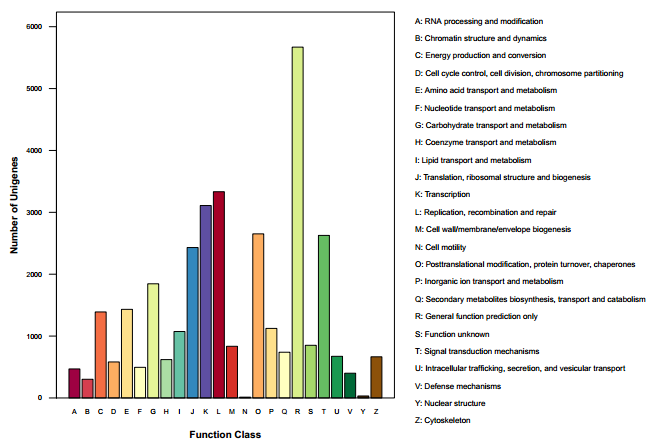


**Supplementary Figure 5**: **COG functional classification of the *P. notoginseng* unigenes.**

**Supplementary Figure 6**: **Classification based on metabolism categories of *P. notoginseng* unigenes.**


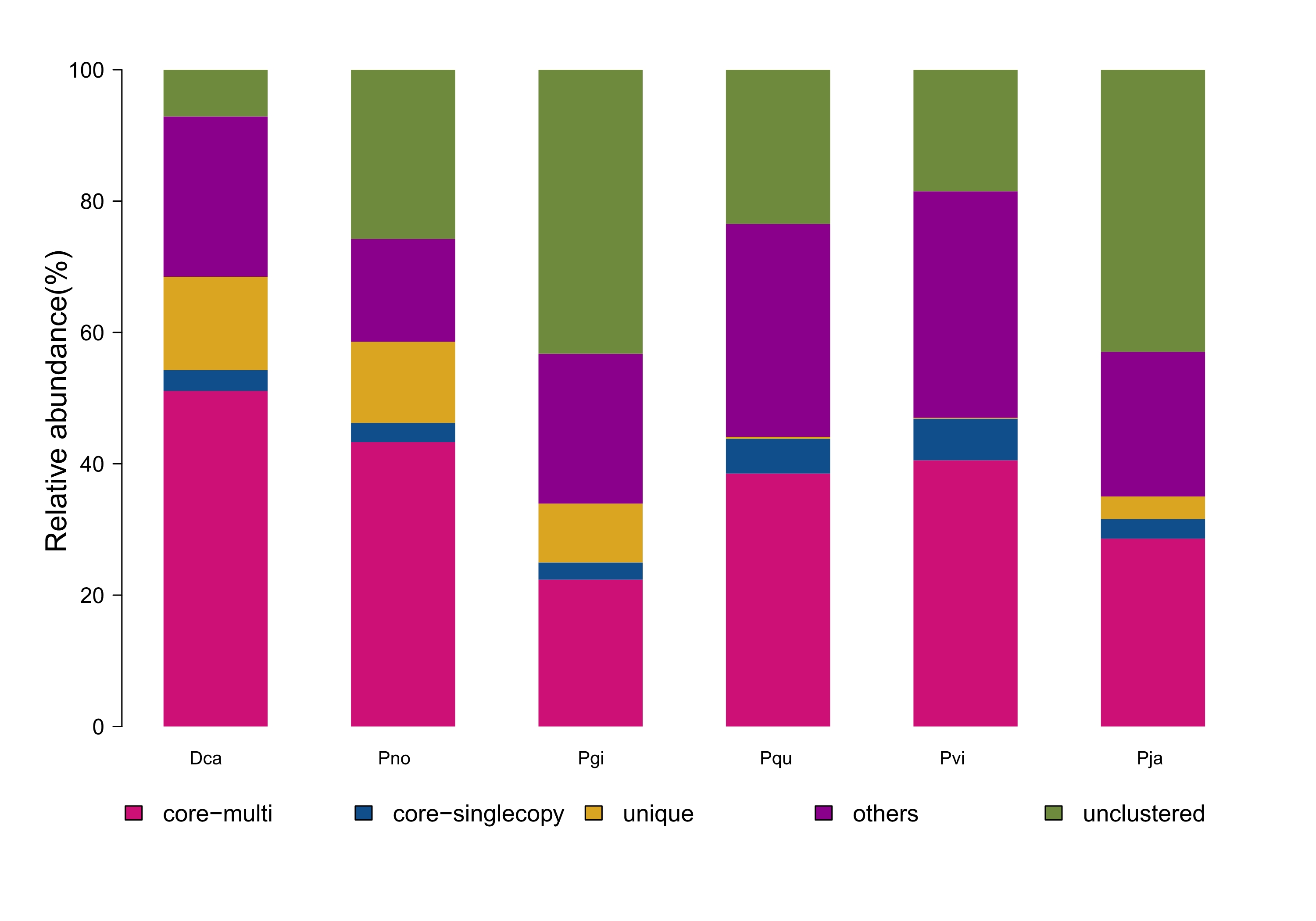


**Supplementary Figure 7**: **Orthologous group assignment among *P. notoginseng* and four other *Panax* species in comparison with *Daucus carota***. Pno, *P. notoginseng*; Pgi, *P. ginseng*; Pqu, *P. quinquefolium*; *Pja, P. japonicas*; Pvi, *P. vietnamensis*; Dca, *D. carota*.


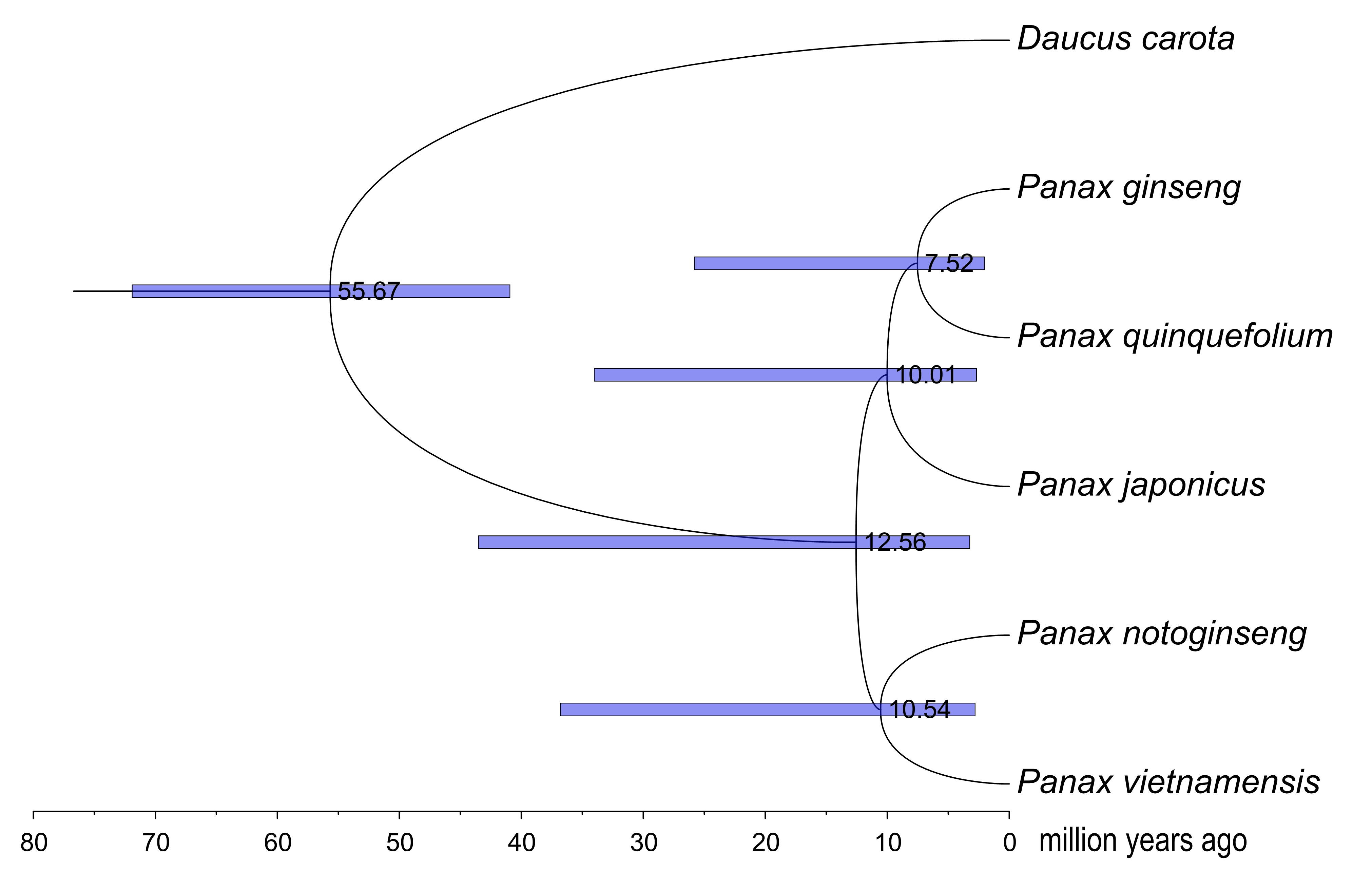


**Supplementary Figure 8**: **Estimation of divergence times among *P. notoginseng*, *P. ginseng, P. quinquefolium, P. japonicas* and *P. vietnamensis* using *D. carota* as outgroup.**

**Supplementary Figure 9**: **Phylogenomic analysis of *P. notoginseng*, *P. ginseng, P. quinquefolium, P. japonicas* and *P. vietnamensis* using *D. carota* as outgroup.** Phylogenetic tree was constructed based on 499 high-quality 1:1 single-copy orthologous genes using RAxML (version 8.1.13) package. The numbers on the nodes represents the bootstrap support values, and the branch lengths are proportional to the levels of species divergence.


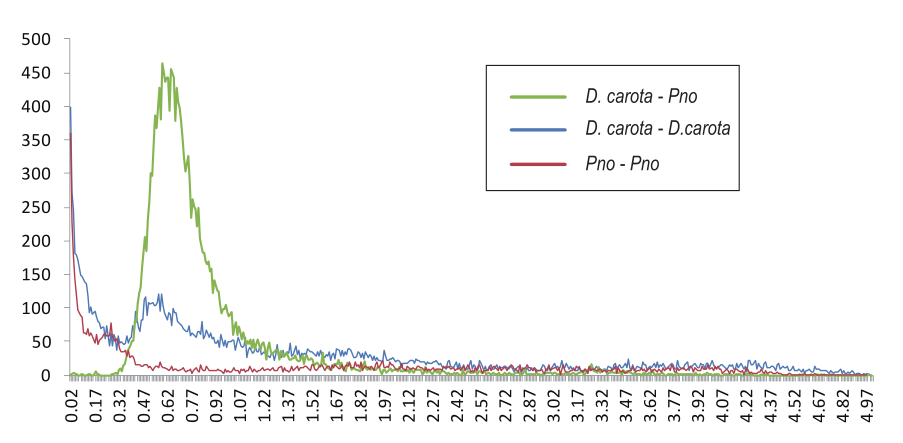


***P. vietnamensis***

***P. japonicas***

***P. quinquefolium***

***P. ginseng***

**Supplementary Figure 10. The *Ks* distribution of paralogous gene pairs between *P. notoginseng* and *D. carota, P. ginseng, P. quinquefolium, P. japonicas*, *P. vietnamensis*, respectively.** Paralogous gene pairs for each species were generated by using a combination of OrthoMCL package (v2.0.9) and blast-based method. *Ks* values between each gene pair are estimated based on the NG (Nei & Gojoberi) method of Yang that implemented in the PAML package. The initial peak (*Ks* < 0.1) is likely to reflect the variation in the birth and death rates of tandem, small-scale, segmental duplications, or alleles, rather than whole genome duplication events.


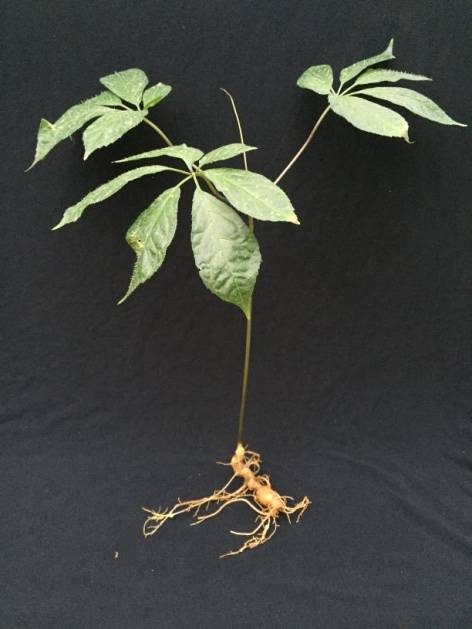

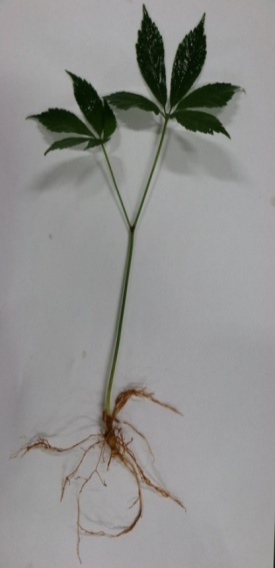

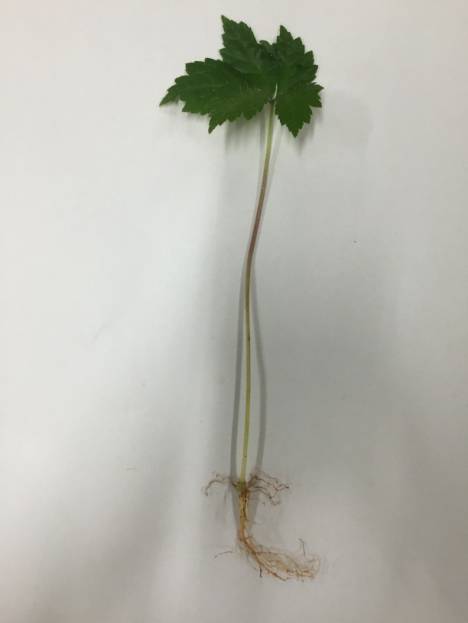


**Supplementary Figure 11: The sample plants of one-year old, two-year old, three -year old *P. notoginseng* for Illumina sequencing.**


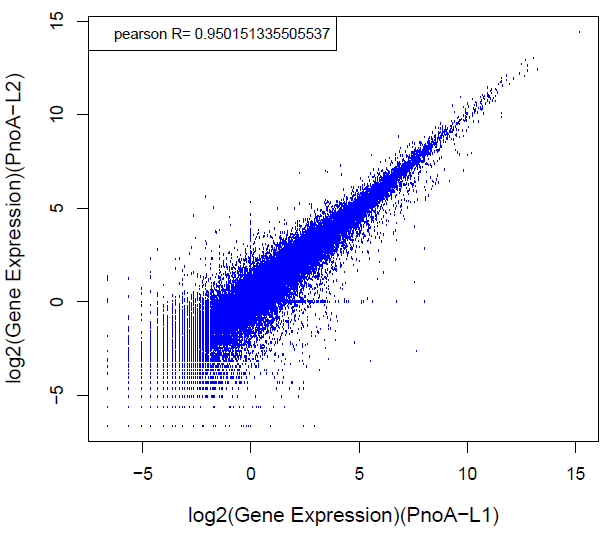

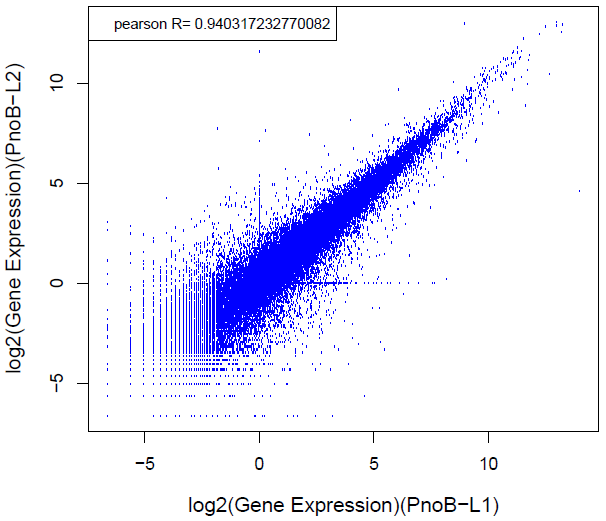

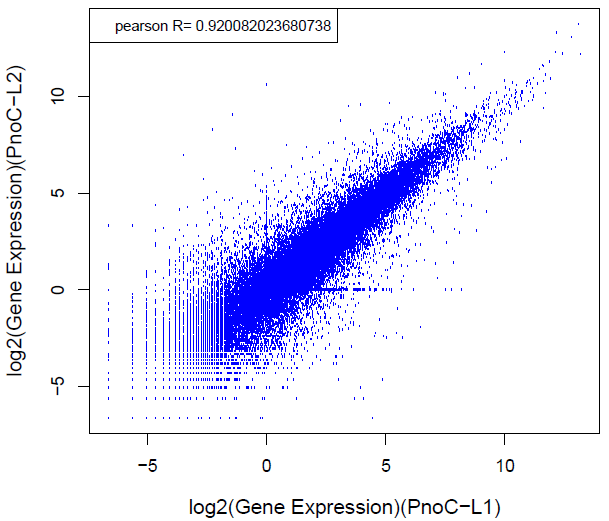


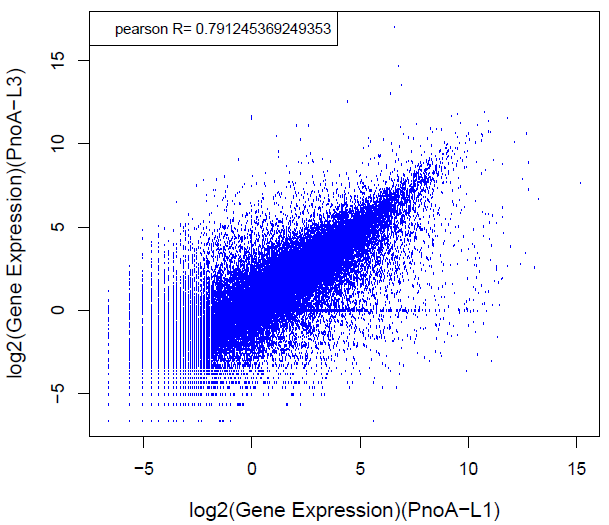

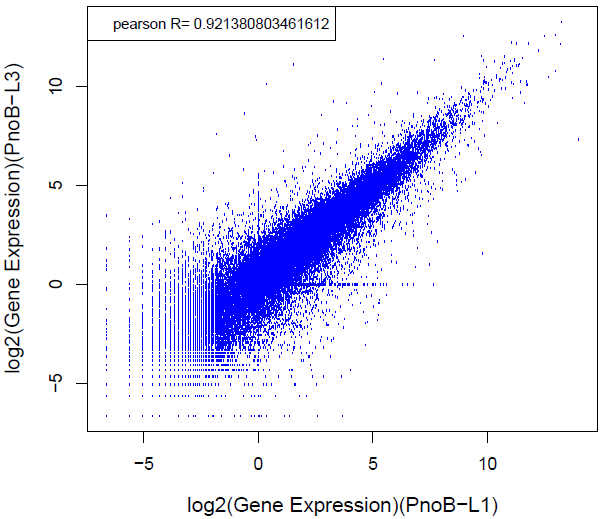

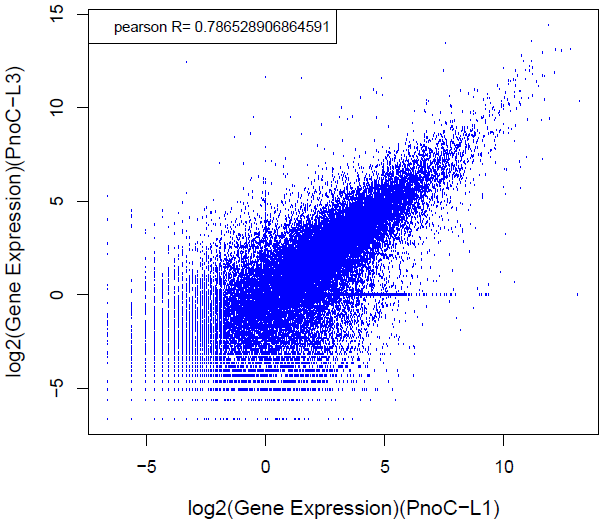


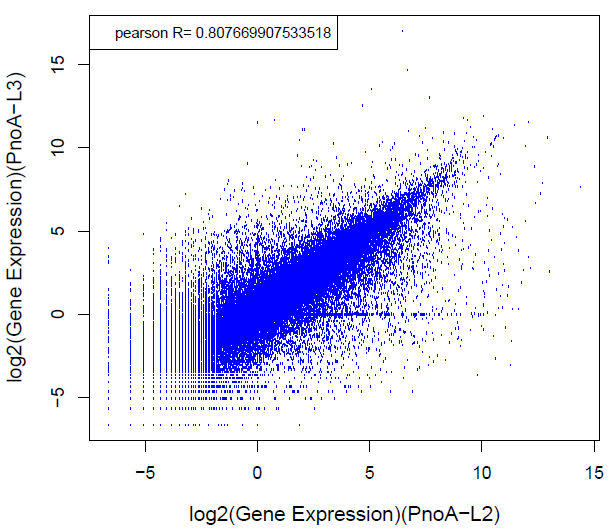

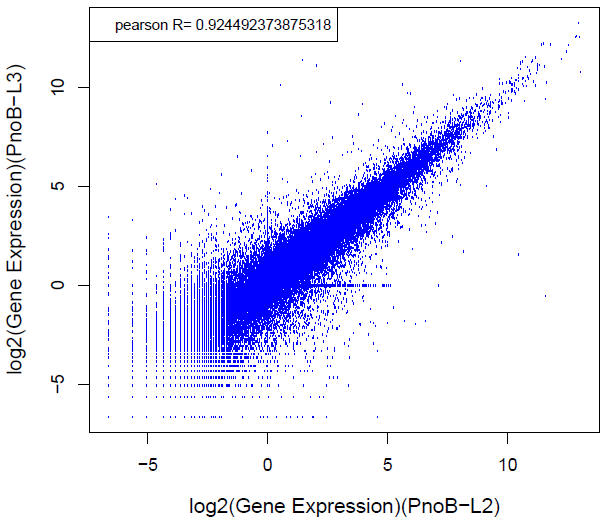

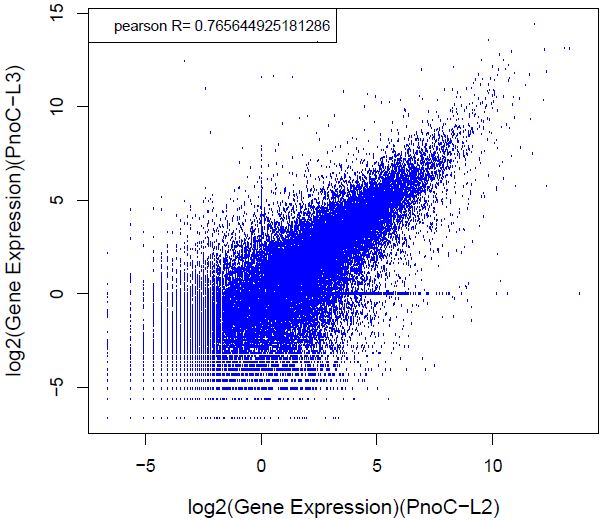


|  | **PnoA-L2** | | **PnoA-L3** |  | **PnoB-L2** | | | **PnoB-L3** | |  | **PnoC-L2** | **PnoC-L3** |
| --- | --- | --- | --- | --- | --- | --- | --- | --- | --- | --- | --- | --- |
| **PnoA-L1** | **0.9502** | **0.7912** | | **PnoB-L1** | | **0.9403** | **0.9214** | | | **PnoC-L1** | **0.9201** | **0.7865** |
| **PnoA-L2** |  | **0.8077** | | **PnoB-L2** | |  | | | **0.9245** | **PnoC-L2** |  | **0.7656** |

**Supplementary Figure 12:** Results of Pearson Correlation Coefficient (PCC) of gene expression levels among the three repeated samples of the 11 tissues from the 1-year old, 2-year old and 3-year old plants of *P. notoginseng*. The results in yellow are displayed in **Supplementary Figure 13**.


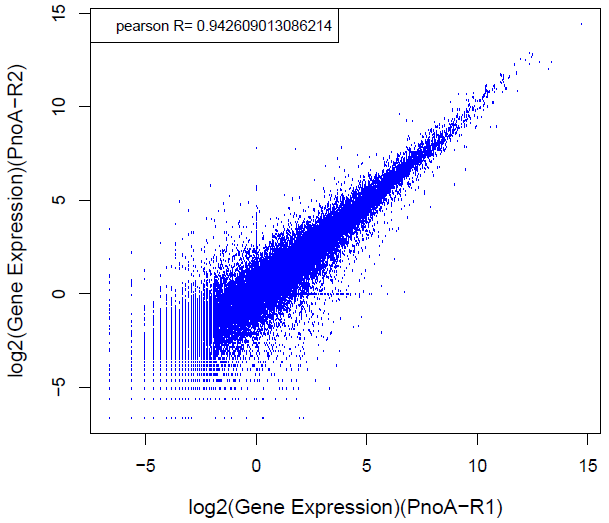

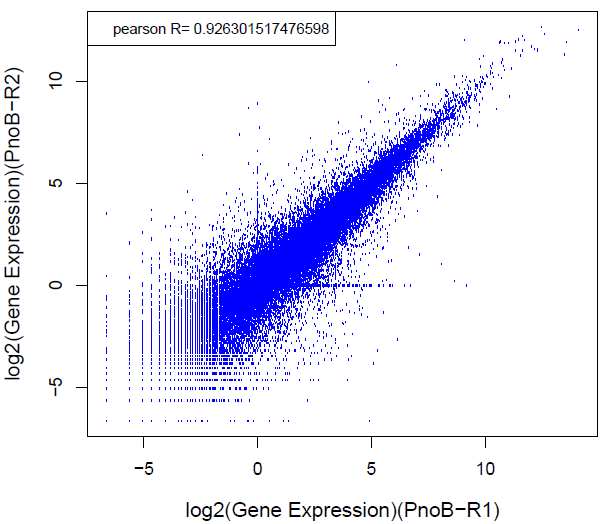

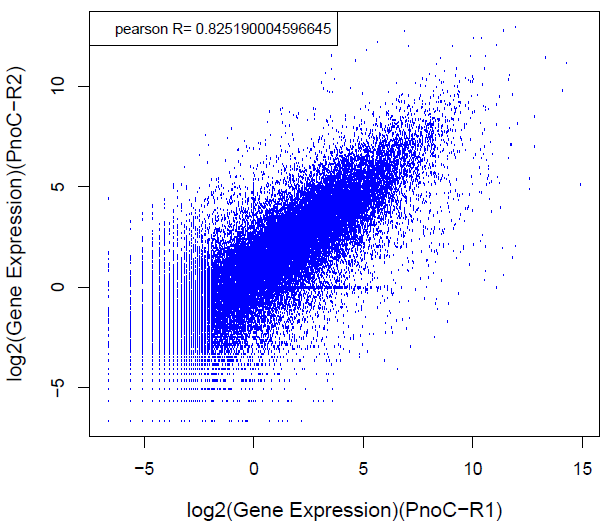


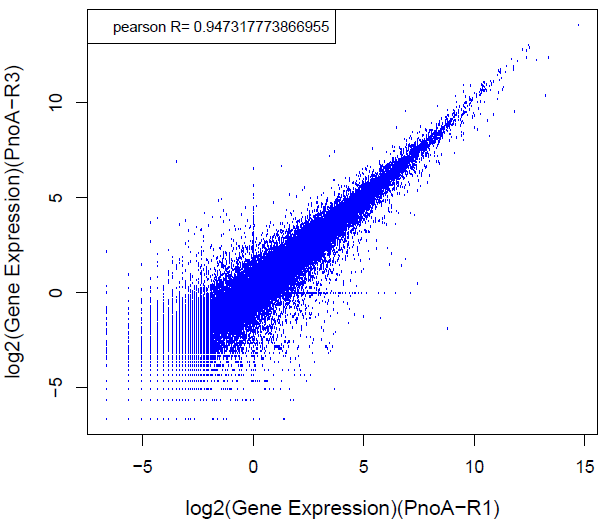

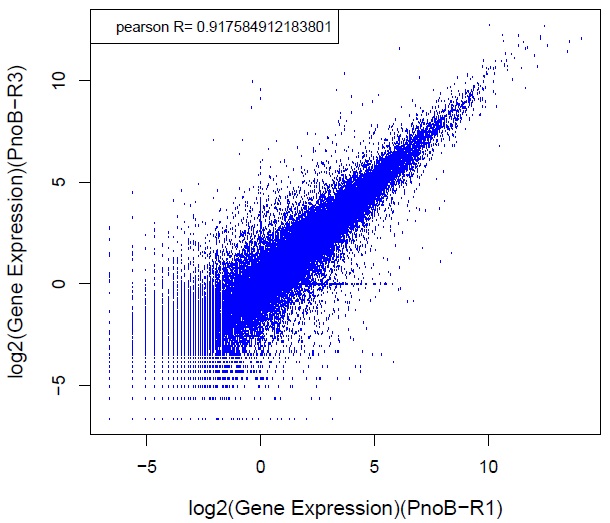

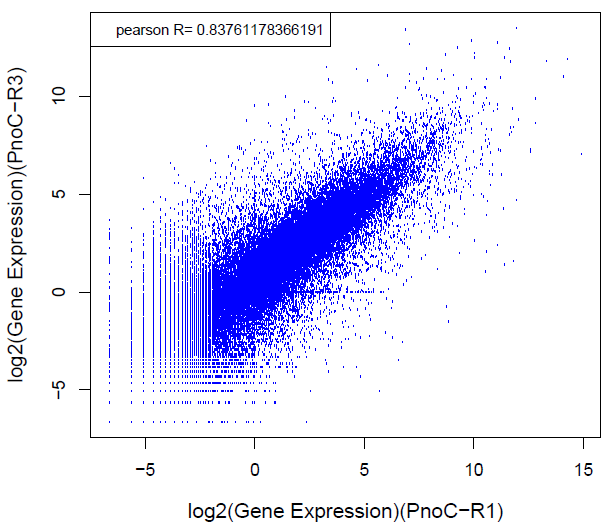


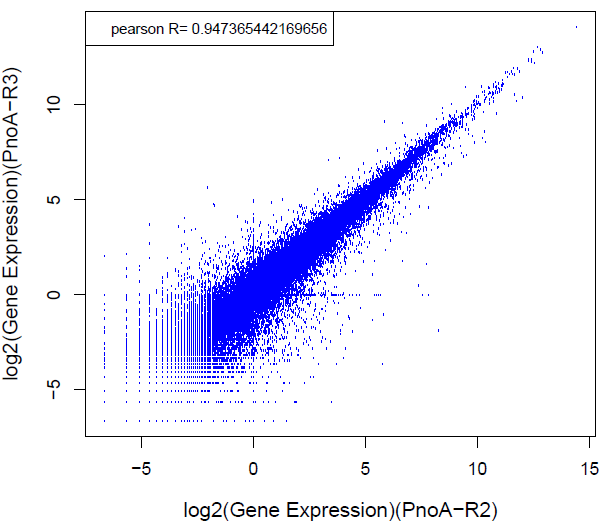

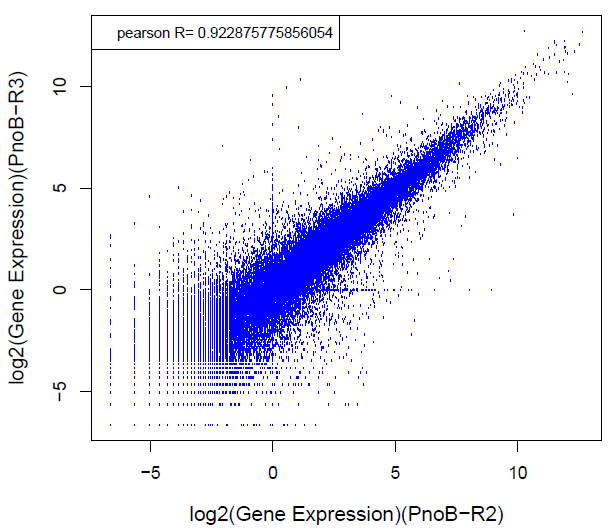

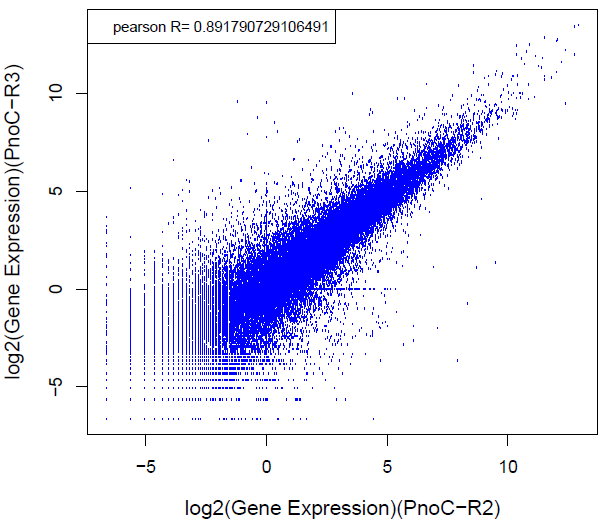


|  | **PnoA-R2** | | **PnoA-R3** |  | **PnoB-R2** | | **PnoB-R3** |  | **PnoC-R2** | | **PnoC-R3** |
| --- | --- | --- | --- | --- | --- | --- | --- | --- | --- | --- | --- |
| **PnoA-R1** | | **0.9426** | **0.9473** | **PnoB-R1** | | **0.9263** | **0.9176** | **PnoC-R1** | | **0.8252** | **0.8376** |
| **PnoA-R2** | | | **0.9474** | **PnoB-R2** | |  | **0.9229** | **PnoC-R2** | |  | **0.8918** |

**Supplementary Figure 12:** Results of Pearson Correlation Coefficient (PCC) of gene expression levels among the three repeated samples of the 11 tissues from the 1-year old, 2-year old and 3-year old plants of *P. notoginseng*. The results in yellow are displayed in **Supplementary Figure 13**.


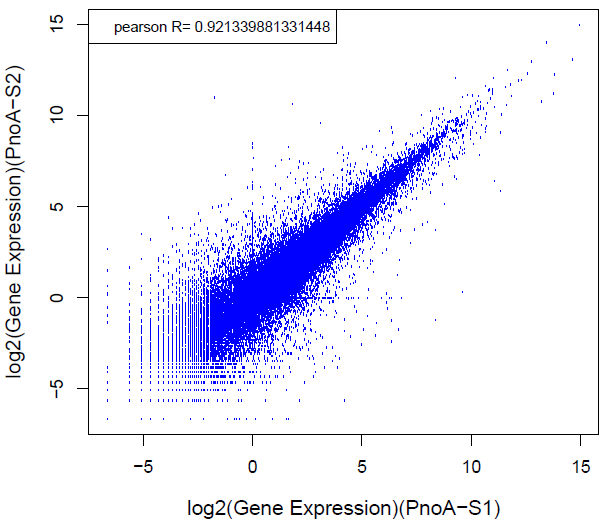

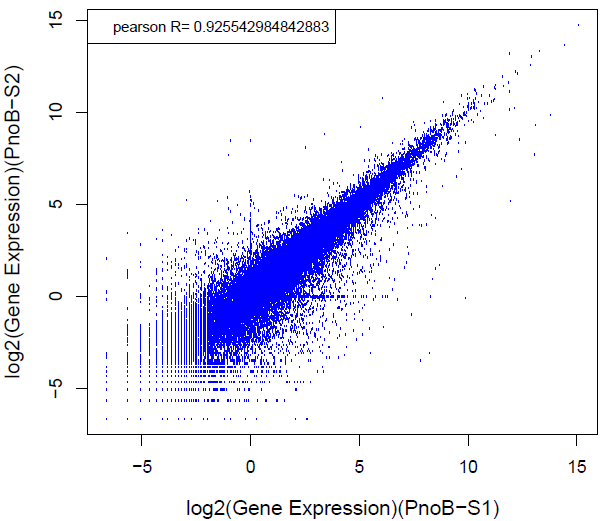

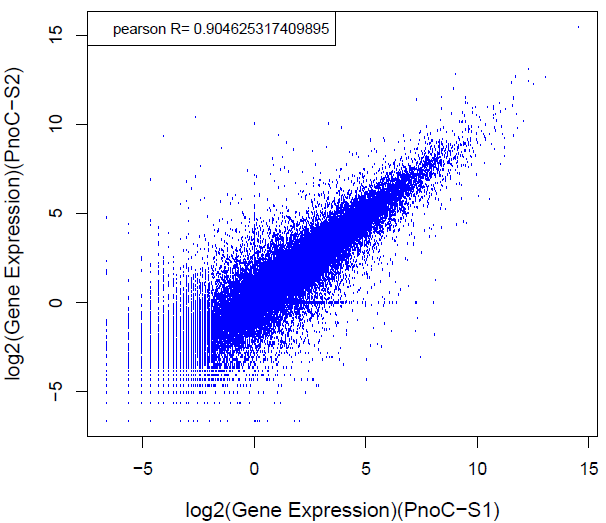


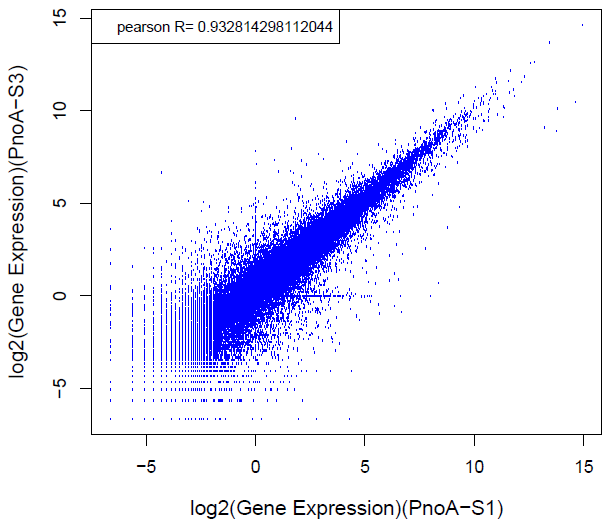

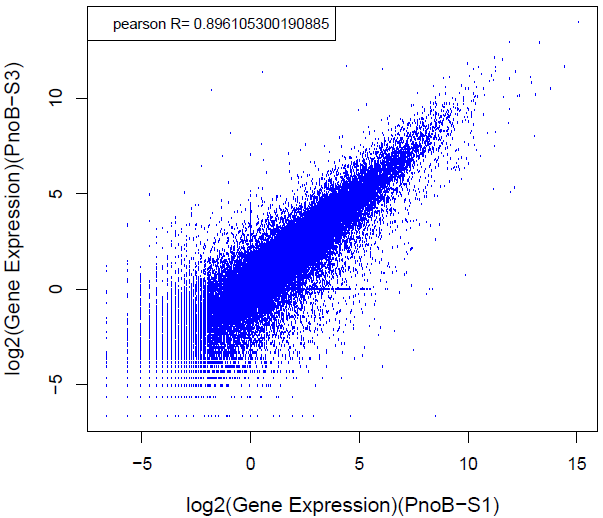

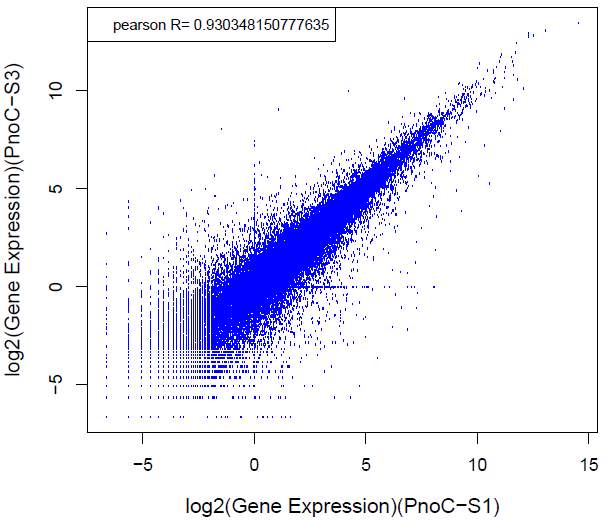


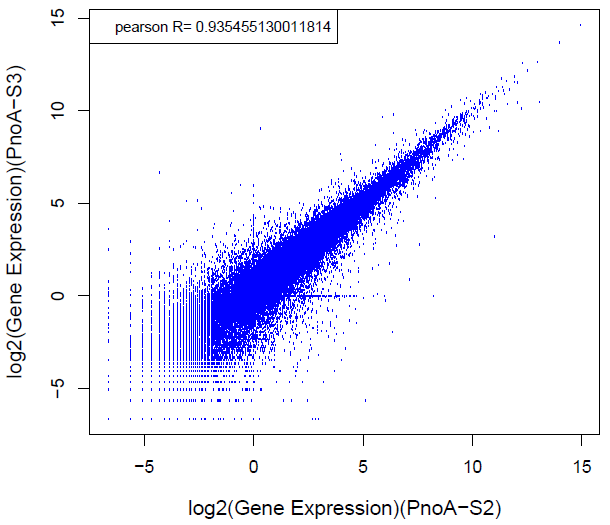

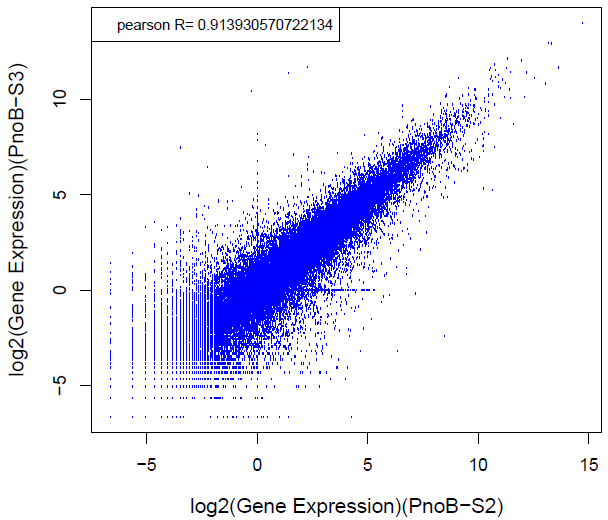

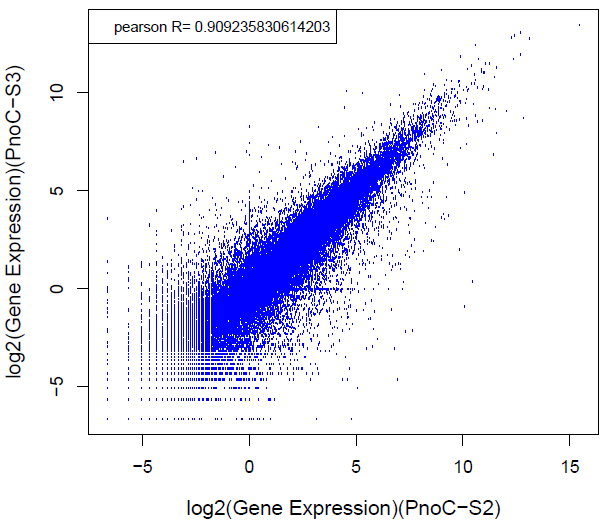


|  | **PnoA-S2** | **PnoA-S3** |  | **PnoB-S2** | **PnoB-S3** |  | **PnoC-S2** | | | **PnoC-S3** |
| --- | --- | --- | --- | --- | --- | --- | --- | --- | --- | --- |
| **PnoA-S1** | **0.9213** | **0.9328** | **PnoB-S1** | **0.9255** | **0.8961** | **PnoC-S1** | | **0.9046** | | **0.9303** |
| **PnoA-S2** | | **0.9355** | **PnoB-S2** |  | **0.9139** | **PnoC-S2** | | |  | **0.9092** |

**Supplementary Figure 12:** Results of Pearson Correlation Coefficient (PCC) of gene expression levels among the three repeated samples of the 11 tissues from the 1-year old, 2-year old and 3-year old plants of *P. notoginseng*. The results in yellow are displayed in **Supplementary Figure 13**.


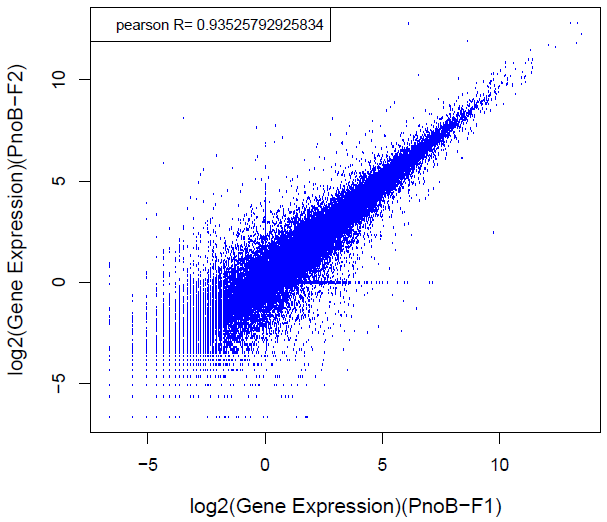

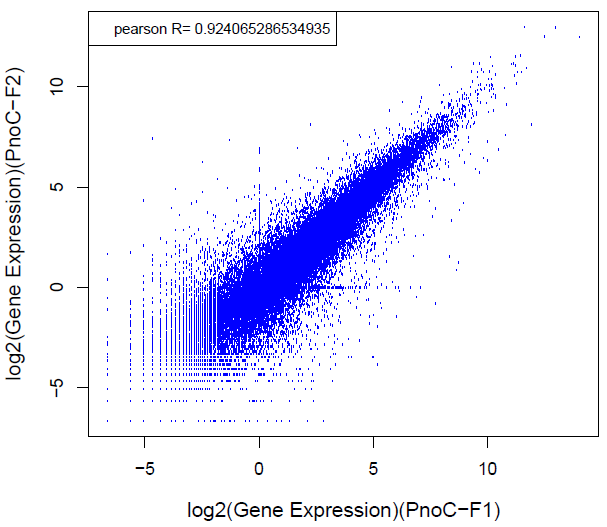


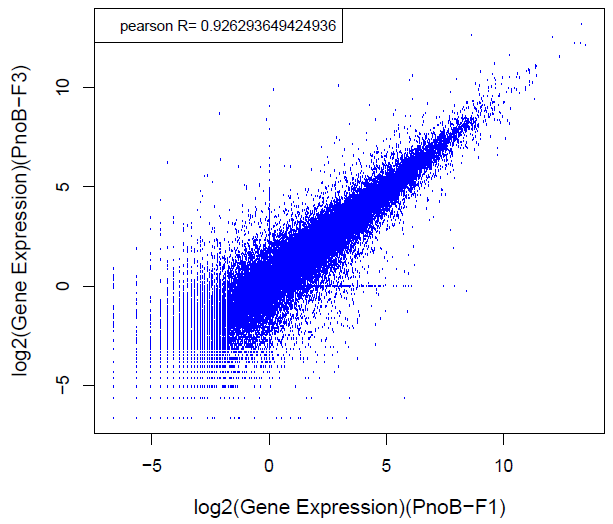

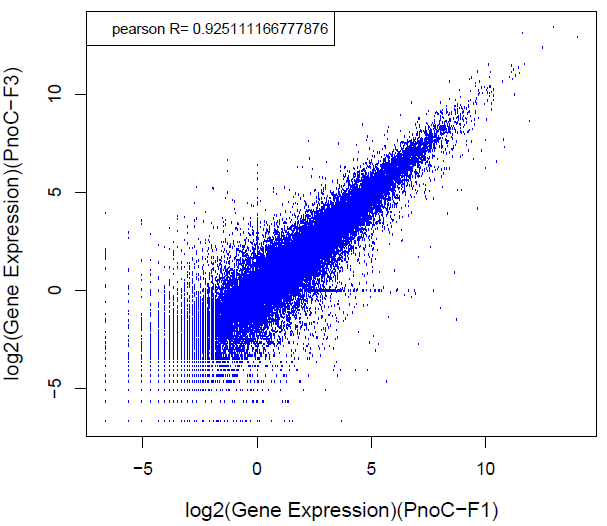


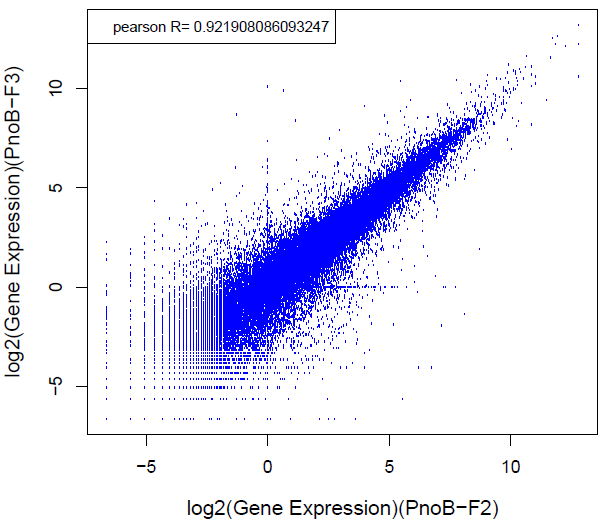

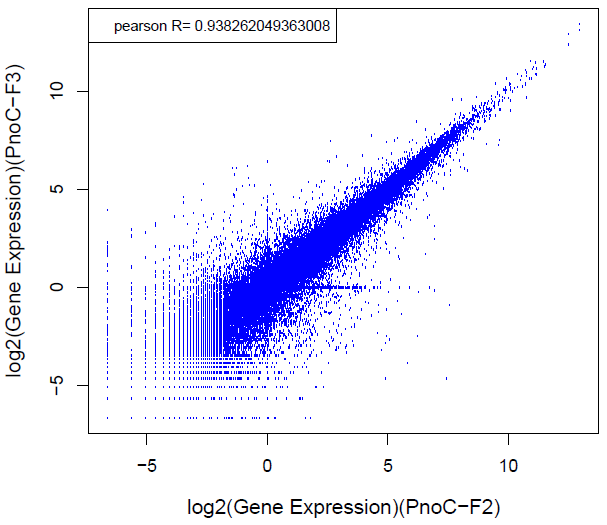


|  | **PnoB-F2** | **PnoB-F3** |  | **PnoC-F2** | **PnoC-F3** |
| --- | --- | --- | --- | --- | --- |
| **PnoB-F1** | **0.9353** | **0.9263** | **PnoC-F1** | **0.9241** | **0.9251** |
| **PnoB-F2** |  | **0.9219** | **PnoC-F2** |  | **0.9383** |

*R represents roots, S represents stems, L represents leaves, F represents flowers, A represents one-year old plant, B represents two-year old plant, and C represents three-year old plant. 1, 2 and 3 represent repeat 1, 2 and 3, respectively.

**Supplementary Figure 12:** Results of Pearson Correlation Coefficient (PCC) of gene expression levels among the three repeated samples of the 11 tissues from the 1-year old, 2-year old and 3-year old plants of *P. notoginseng*. The results in yellow are displayed in **Supplementary Figure 13.**

**Supplementary Figure 13: A heat map of differentially expressed genes involved in the ginsenoside biosynthesis among the eleven developmental tissues collected from the one-year old, two-year old and three-year old plants of *P. notoginseng*.** (A) The longest sequence for each gene family was selected to evaluate expression levels by TPM, further centered and plotted by using R language; (B) the top gene expression level for each gene family was detected by using the above-mentioned method.

**
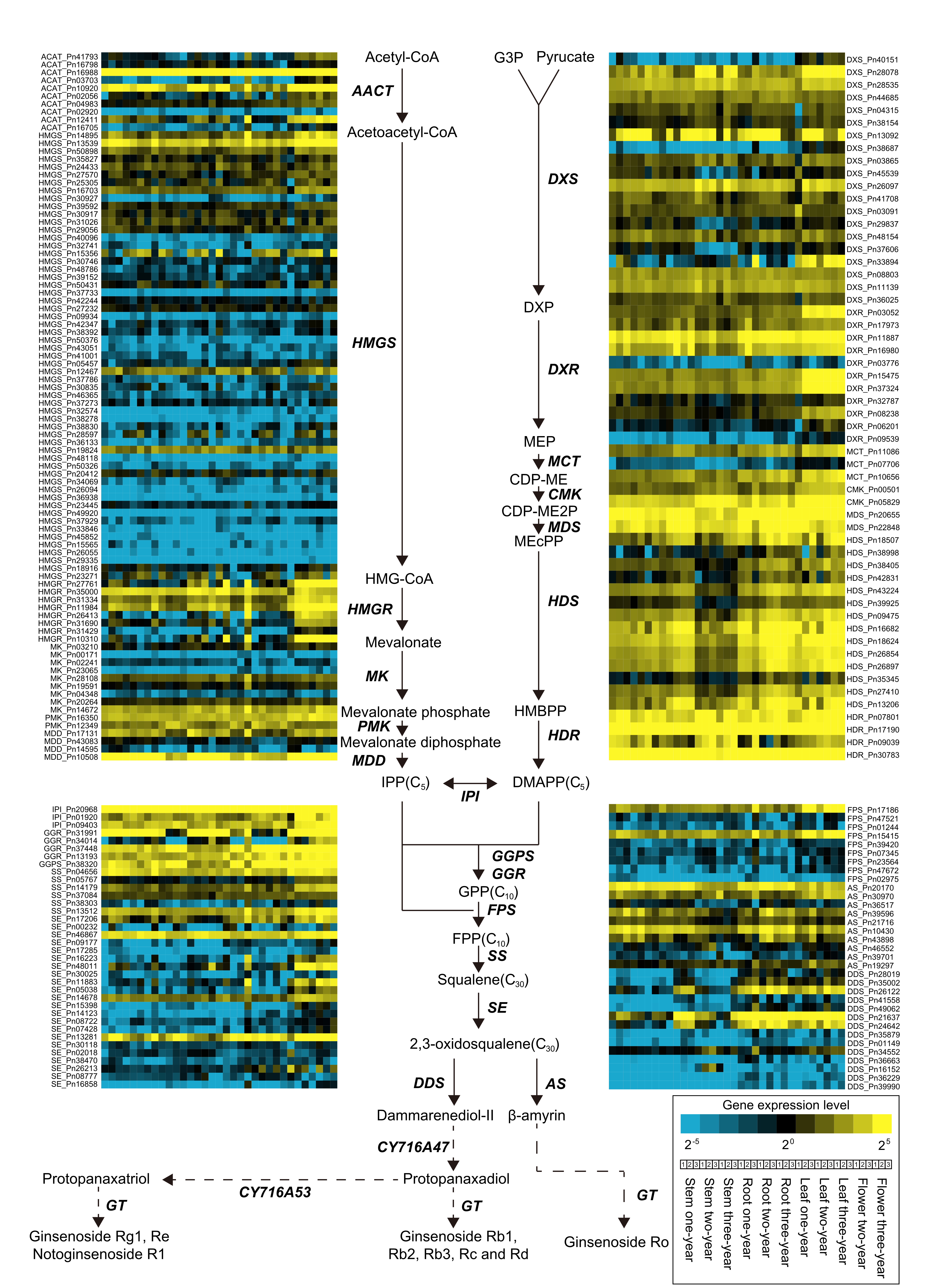
**

**Supplementary Figure 14: A heat map of differentially expressed genes involved in the ginsenoside biosynthesis among the eleven developmental tissues collected from the one-year old, two-year old and three-year old plants of *P. notoginseng*.** The heat map was drawn based on TPM values of the three biological repeats, of which high to low expression levels were indicated from yellow to blue in color; 33 boxes represent different tissues collected from the one-year old, two-year old and three-year old plants of *P. notoginseng*.


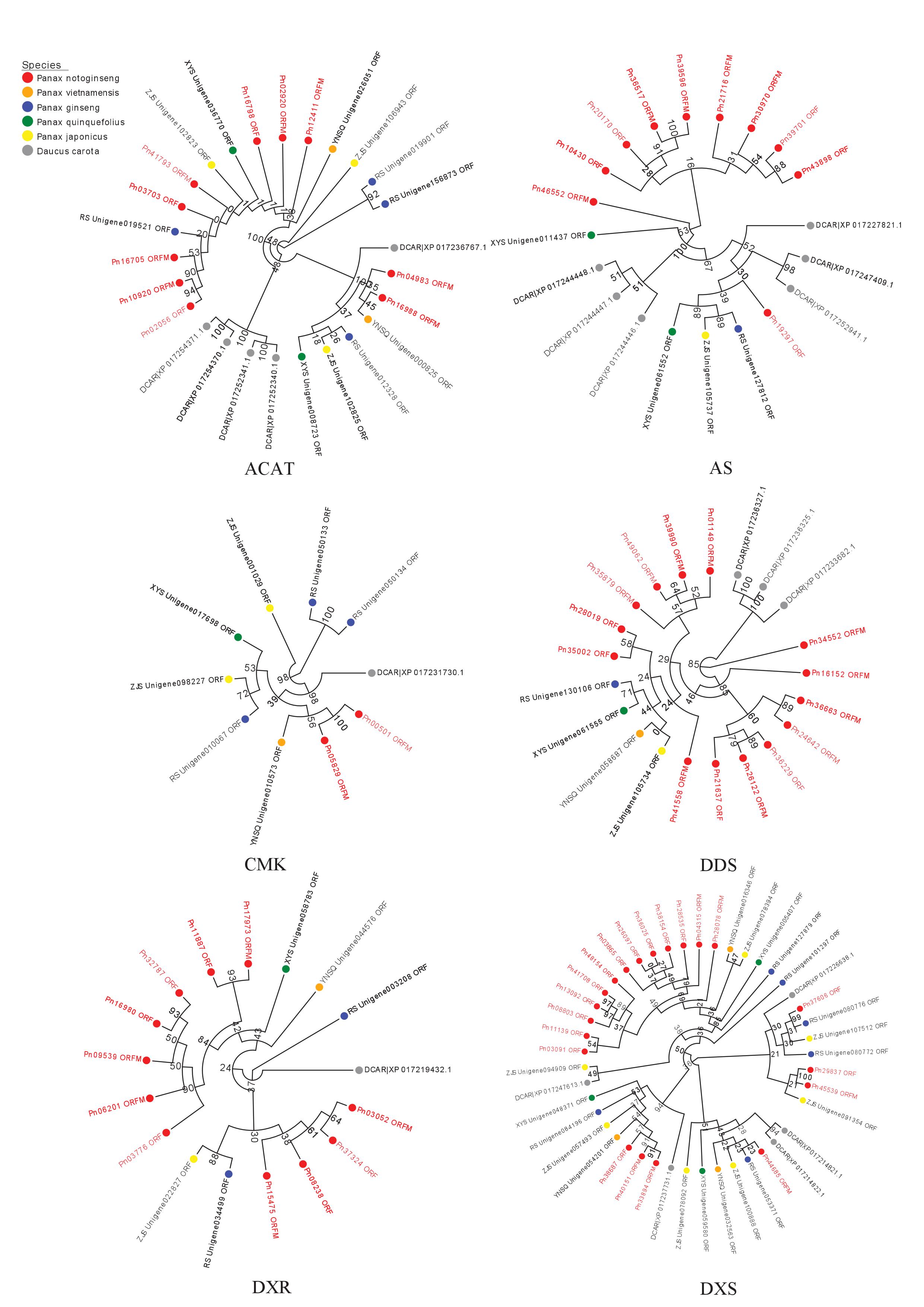


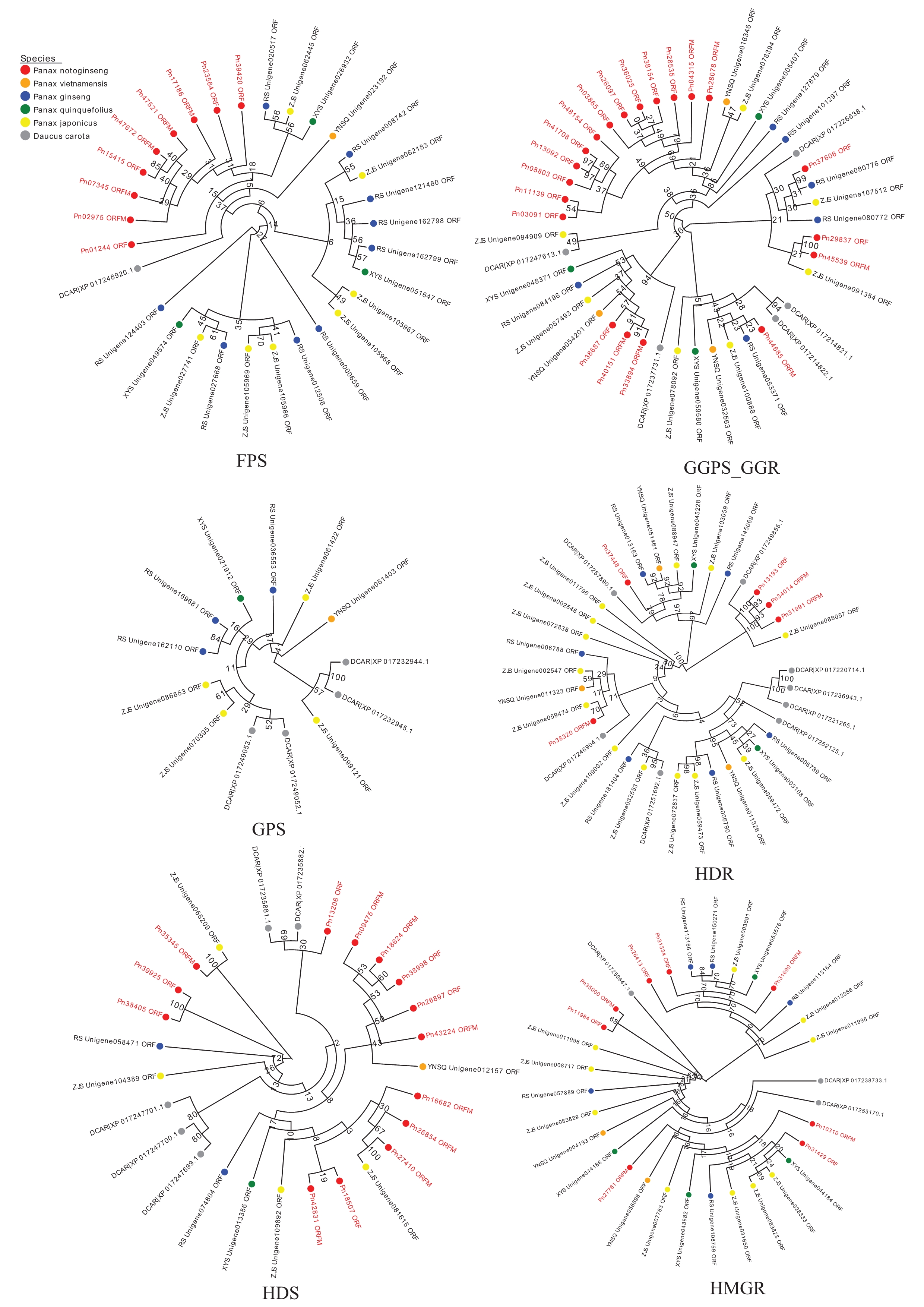


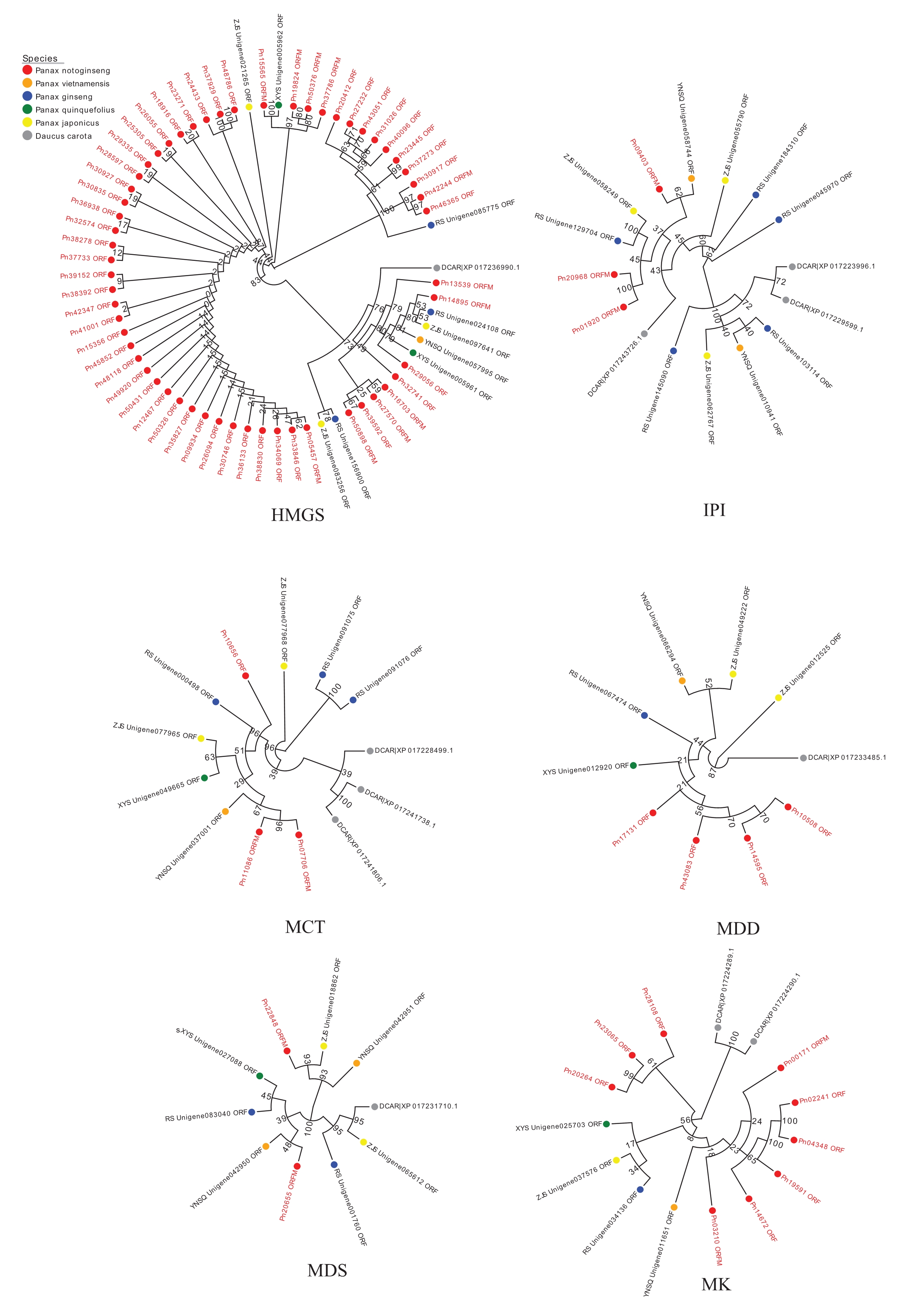


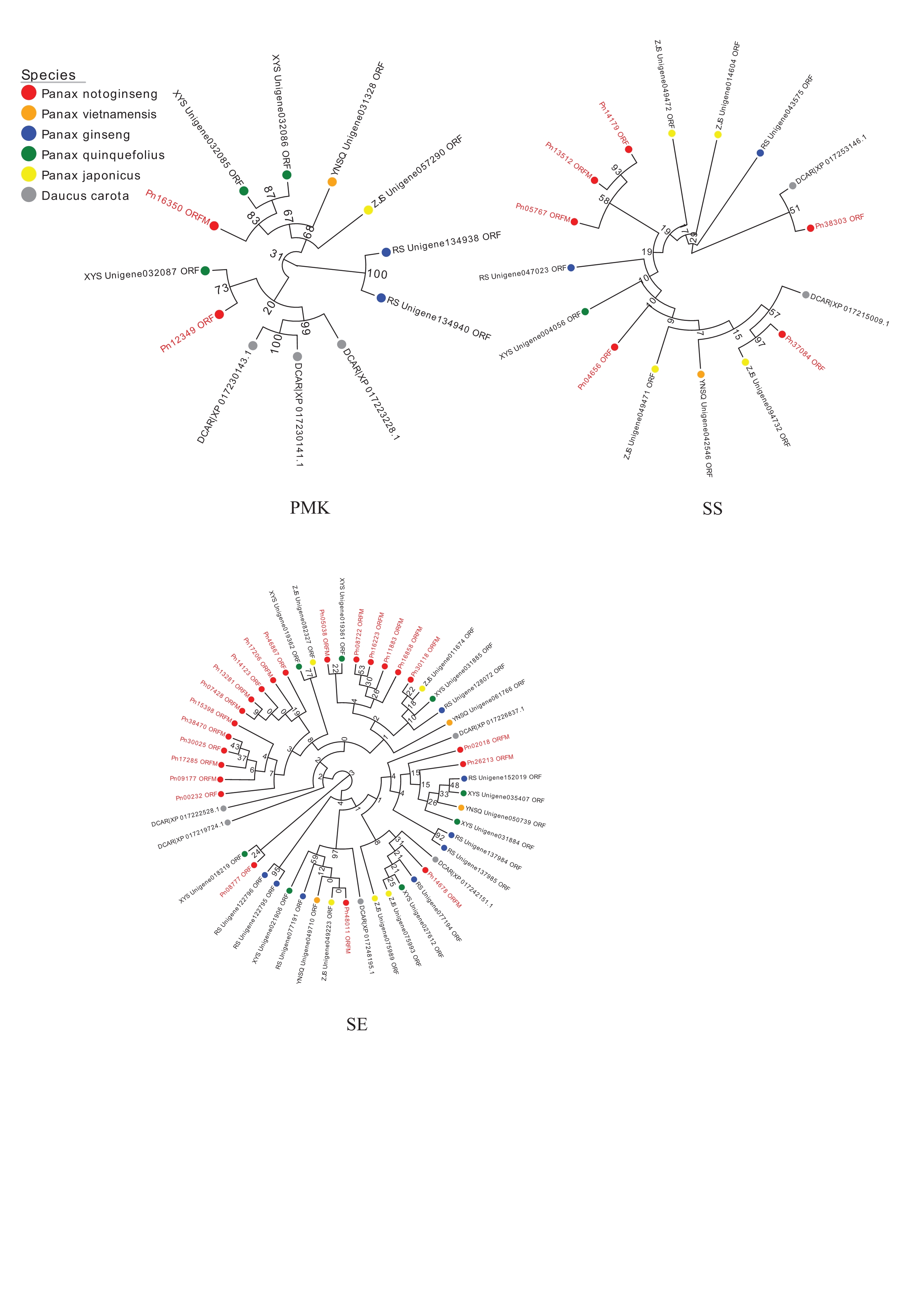


**Supplementary Figure 15: Neighbor-joining (NJ) phylogenetic tree of the 21 genes involved in ginsenoside biosynthesis of *P. notoginseng*, *P. ginseng, P. quinquefolium, P. japonicas* and *P. vietnamensis* and *D. carota*.** The NJ tree was constructed using TreeBeST (Version: 1.9.2) with the JTT model and 1000 bootstrap replicates. The bootstrap support values that were divided by 100 are shown on the tree.


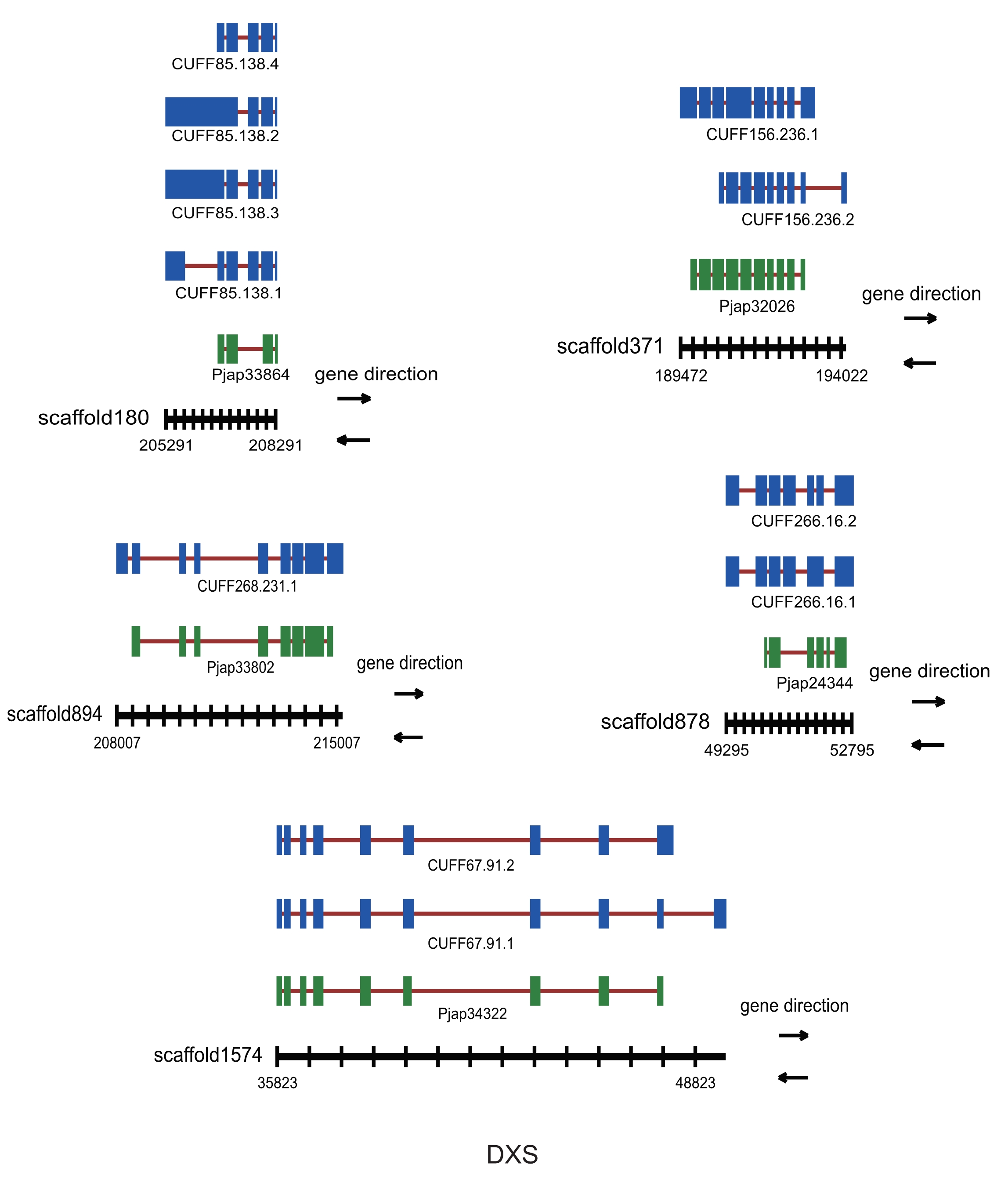


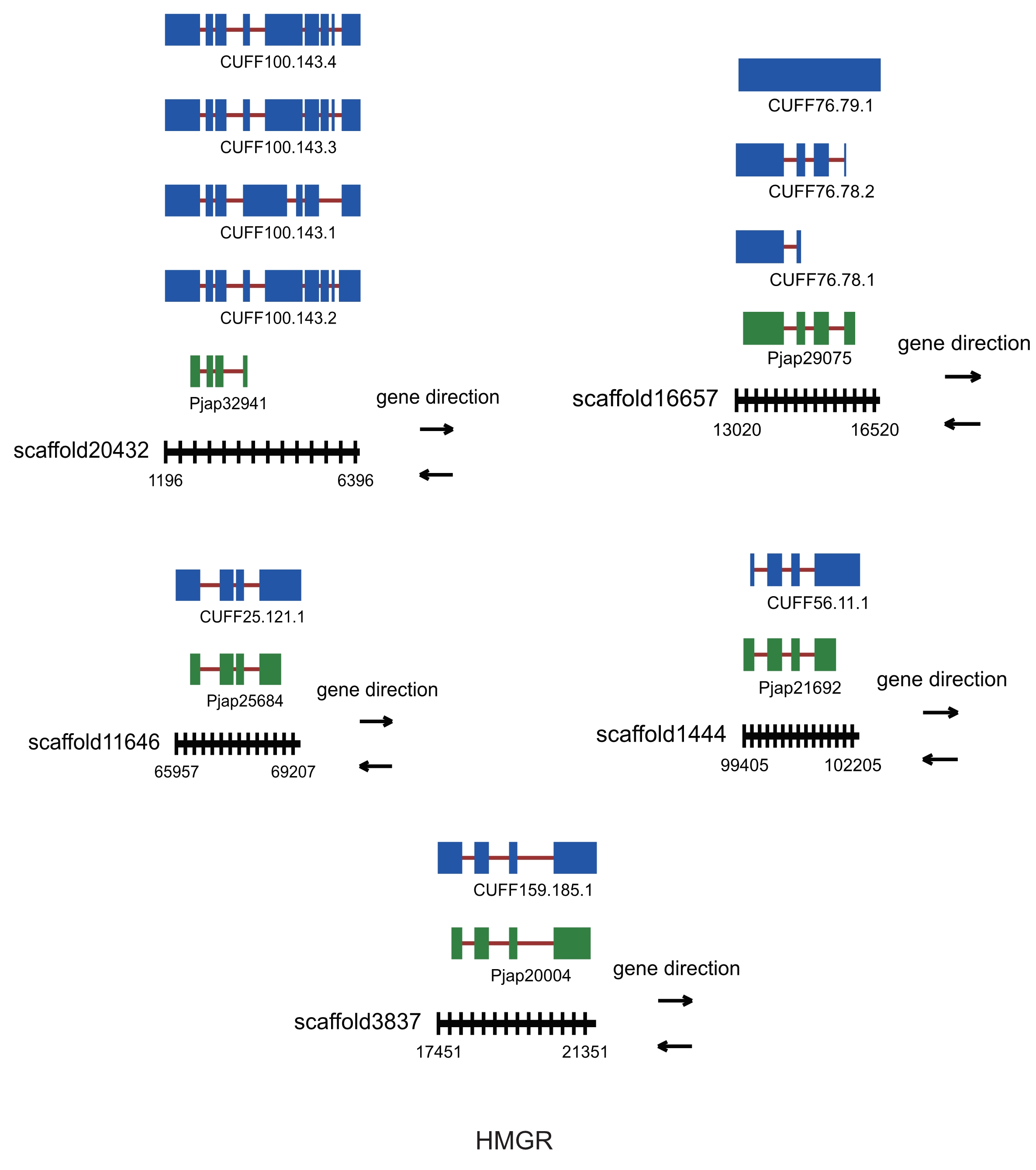


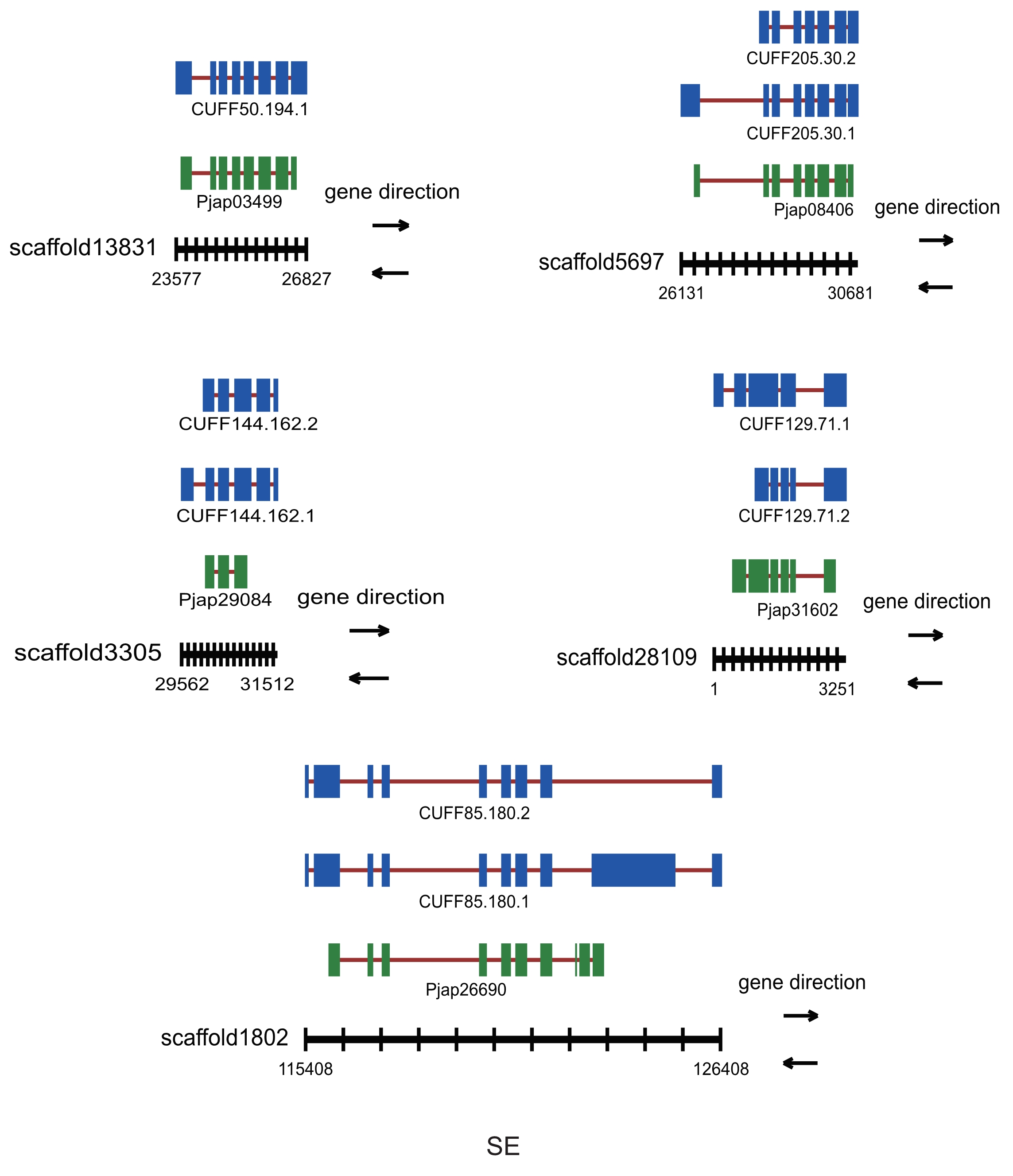


**Supplementary Figure 16: The AS events of genes involved in the ginsenoside biosynthesis in *P. notoginseng*.** The green bar represents gene model, blue bar represents AS events from cufflinks, black bar represents scaffold locations and the arrow represents gene direction.

**Supplementary Tables**

**Supplementary Table 1**: **Summary of RNA sequencing (RNA-Seq) of five tissues of *P. notoginseng*.**

| **Tissues** | **Read length (bp)** | **Number of reads (M*)** | **Clean data (Gb)** |
| --- | --- | --- | --- |
| Root | 100 | ~ 25.5 | ~5.1 |
| Stem | 100 | ~ 32.4 | ~ 6.4 |
| Leaf | 100 | ~ 35.2 | ~7.0 |
| Flower | 100 | ~ 36.6 | ~ 7.3 |
| Rhizome | 100 | ~ 25.5 | ~ 5.1 |
| **Total** |  | **~ 155.2** | **~ 30.9** |

**Supplementary Table 2**: **Summary of SMRT unigenes of *P. notoginseng* after the correction by Illumina short reads.**

|  | **SMRT Unigenes** | | **Illumina Unigenes** | | | |
| --- | --- | --- | --- | --- | --- | --- |
|  | **Length(bp)** | **Number** | | **Length(bp) Number** | | |
| **Maximum Length** | 20,087 |  | 16,703 | |  | |
| **N10** | 4,546 | 2,225 | 3,848 | | 2,566 | |
| **N20** | 3,911 | 5,241 | 2,937 | | 6,367 | |
| **N30** | 3,537 | 8,629 | 2,361 | | 11,194 | |
| **N40** | 3,177 | 12,410 | 1,923 | | 17,149 | |
| **N50** | 2,967 | 16,524 | 1,532 | | 24,504 | |
| **N60** | 2,775 | 20,910 | 1,155 | | 34,009 | |
| **N70** | 2,156 | 26,011 | 802 | | 47,126 | |
| **N80** | 1,823 | 32,404 | 516 | | 66,830 | |
| **N90** | 1,552 | 39,862 | 317 | | 98,483 | |
| **Total** | 126,090,472 | 51,027 | 126,507,649 | | 149,827 | |
| **number>=100bp** |  | 51,027 |  | | 149,827 | |
| **number>=2000bp** |  | 28,539 |  | | 15,935 | |
| **GC content** | 40.6% |  | 38.89% | | |  |

**Supplementary Table 3**: **Summary of annotation of *P. notoginseng.***

| **Database** | **Number** |
| --- | --- |
| **All unigene** | 51,040 |
| **All annotated** | 49,632 |
| **Nr** | 49,380 |
| **Nt** | 39,348 |
| **Swiss-Prot** | 36,024 |
| **KEGG** | 33,817 |
| **COG** | 24,331 |
| **GO** | 38,298 |

**Supplementary Table 4**: **The summary of COG function annotation of unigenes in *P. notoginseng.***

|  | Functional-Categories | No. Transcript |
| --- | --- | --- |
| A | RNA processing and modification | 469 |
| B | Chromatin structure and dynamics | 302 |
| C | Energy production and conversion | 1,390 |
| D | Cell cycle control, cell division, chromosome partitioning | 582 |
| E | Amino acid transport and metabolism | 1,432 |
| F | Nucleotide transport and metabolism | 495 |
| G | Carbohydrate transport and metabolism | 1,845 |
| H | Coenzyme transport and metabolism | 620 |
| I | Lipid transport and metabolism | 1,074 |
| J | Translation, ribosomal structure and biogenesis | 2,431 |
| K | Transcription | 3,110 |
| L | Replication, recombination and repair | 3,333 |
| M | Cell wall/membrane/envelope biogenesis | 835 |
| N | Cell motility | 13 |
| O | Posttranslational modification, protein turnover, chaperones | 2,652 |
| P | Inorganic ion transport and metabolism | 1124 |
| Q | Secondary metabolites biosynthesis, transport and catabolism | 739 |
| R | General function prediction only | 5,670 |
| S | Function unknown | 851 |
| T | Signal transduction mechanisms | 2,627 |
| U | Intracellular trafficking, secretion, and vesicular transport | 673 |
| V | Defense mechanisms | 400 |
| Y | Nuclear structure | 32 |
| Z | Cytoskeleton | 665 |

**Supplementary Table 5**: **The summary of predicted CDS in *P. notoginseng.***

| **Method** | **ORF** |
| --- | --- |
| Homolog | 44,303 |
| ESTScan | 329 |
| Markov_5 | 3,688 |
| Total | 48,320 |

**Supplementary Table 6**: **Summary of pathway assignment based on KEGG.**

| **KEGG pathways** | **Number of Unigenes** |
| --- | --- |
| **Metabolism** |  |
| Metabolism of cofactors and vitamins | 1219 |
| Amino acid metabolism | 3094 |
| Nucleotide metabolism | 2061 |
| Metabolism of terpenoids and polyketides | 1739 |
| Glycan biosynthesis and metabolism | 748 |
| Lipid metabolism | 1691 |
| Xenobiotics biodegradation and metabolism | 1161 |
| Energy metabolism | 1748 |
| Carbohydrate metabolism | 4347 |
| Metabolism of other amino acids | 829 |
| Biosynthesis of other secondary metabolites | 788 |
| **Genetic Information Processing** |  |
| Replication and repair | 704 |
| Translation | 1074 |
| Transcription | 428 |
| Folding, sorting and degradation | 1150 |
| **Organismal Systems** |  |
| Nervous system | 507 |
| Excretory system | 133 |
| Sensory system | 32 |
| Digestive system | 180 |
| Circulatory system | 29 |
| Endocrine system | 738 |
| Immune system | 1032 |
| Development | 121 |
| Environmental adaptation | 125 |
| **Cellular Processes** |  |
| Cell growth and death | 1068 |
| Cell motility | 162 |
| Transport and catabolism | 1011 |
| Cellular community - eukaryotes | 348 |
| **Environmental Information Processing** |  |
| Signal transduction | 2010 |
| Signaling molecules and interaction | 3 |
| Membrane transport | 563 |
| **Total** | 30,843 |

**Supplementary Table 7: The quality assessment of transcript assembly by DETONATE.**

|  | **Illumina** | **SMRT** |
| --- | --- | --- |
| Score | -25093185054.93 | -23383278787.83 |
| BIC_penalty | -1404994.56 | -478631.01 |
| Prior_score_on_contig_lengths_(f_function_canceled) | -403350.05 | -305763.39 |
| Prior_score_on_contig_sequences | -175376840.45 | -174800151.70 |
| Data_likelihood_in_log_space_without_correction | -24916480191.30 | -23207824135.92 |
| Correction_term_(f_function_canceled) | -480321.42 | -129894.19 |
| Number_of_contigs | 149827 | 51040 |
| Expected_number_of_aligned_reads_given_the_data | 56091797.34 | 64951358.65 |
| Number_of_contigs_smaller_than_expected_read/fragment_length | 46992 | 45 |
| Number_of_contigs_with_no_read_aligned_to | 32975 | 2126 |
| Maximum_data_likelihood_in_log_space | -24911547705.39 | -23204483402.25 |
| Number_of_alignable_reads | 57154527 | 67036873 |
| Number_of_alignments_in_total | 90661612 | 248686999 |

**Supplementary Table 8: The quality assessment of transcript assembly using contig Ex90N50 statistic.**

| **Illumina** | | | **SMRT** | | |
| --- | --- | --- | --- | --- | --- |
| Ex | **ExN50** | **num_transcripts** | **Ex** | **ExN50** | **num_transcripts** |
| 1 | 552 | 1 | 1 | 501 | 1 |
| 2 | 552 | 2 | 2 | 501 | 2 |
| 3 | 1130 | 3 | 3 | 501 | 3 |
| 4 | 1039 | 5 | 4 | 2843 | 5 |
| 5 | 913 | 7 | 5 | 676 | 6 |
| 6 | 938 | 9 | 6 | 1146 | 8 |
| 7 | 913 | 11 | 7 | 1639 | 11 |
| 8 | 801 | 14 | 8 | 1183 | 13 |
| 9 | 1130 | 17 | 9 | 1506 | 15 |
| 10 | 1130 | 20 | 10 | 1383 | 18 |
| 11 | 1186 | 24 | 11 | 1146 | 22 |
| 12 | 1186 | 29 | 12 | 1183 | 25 |
| 13 | 1385 | 33 | 13 | 1382 | 29 |
| 14 | 1328 | 39 | 14 | 1146 | 33 |
| 15 | 1385 | 45 | 15 | 1146 | 38 |
| 16 | 1328 | 52 | 16 | 1133 | 44 |
| 17 | 1328 | 60 | 17 | 1133 | 49 |
| 18 | 1231 | 70 | 18 | 1124 | 55 |
| 19 | 1202 | 81 | 19 | 1133 | 62 |
| 20 | 1200 | 93 | 20 | 1104 | 70 |
| 21 | 1202 | 107 | 21 | 1119 | 79 |
| 22 | 1228 | 123 | 22 | 1124 | 88 |
| 23 | 1231 | 140 | 23 | 1129 | 99 |
| 24 | 1328 | 160 | 24 | 1129 | 110 |
| 25 | 1346 | 182 | 25 | 1119 | 123 |
| 26 | 1346 | 206 | 26 | 1129 | 137 |
| 27 | 1358 | 233 | 27 | 1128 | 153 |
| 28 | 1366 | 262 | 28 | 1124 | 169 |
| 29 | 1375 | 293 | 29 | 1158 | 186 |
| 30 | 1401 | 328 | 30 | 1139 | 205 |
| 31 | 1401 | 366 | 31 | 1147 | 225 |
| 32 | 1358 | 408 | 32 | 1196 | 246 |
| 33 | 1361 | 453 | 33 | 1158 | 269 |
| 34 | 1363 | 502 | 34 | 1139 | 294 |
| 35 | 1355 | 556 | 35 | 1146 | 321 |
| 36 | 1358 | 614 | 36 | 1166 | 349 |
| 37 | 1357 | 678 | 37 | 1209 | 378 |
| 38 | 1346 | 745 | 38 | 1196 | 409 |
| 39 | 1364 | 818 | 39 | 1218 | 441 |
| 40 | 1385 | 895 | 40 | 1226 | 476 |
| 41 | 1390 | 980 | 41 | 1268 | 514 |
| 42 | 1401 | 1070 | 42 | 1268 | 554 |
| 43 | 1408 | 1168 | 43 | 1272 | 598 |
| 44 | 1401 | 1274 | 44 | 1301 | 645 |
| 45 | 1401 | 1387 | 45 | 1319 | 694 |
| 46 | 1411 | 1509 | 46 | 1337 | 747 |
| 47 | 1435 | 1639 | 47 | 1333 | 803 |
| 48 | 1457 | 1778 | 48 | 1360 | 864 |
| 49 | 1457 | 1927 | 49 | 1332 | 928 |
| 50 | 1473 | 2085 | 50 | 1363 | 995 |
| 51 | 1477 | 2254 | 51 | 1379 | 1068 |
| 52 | 1500 | 2434 | 52 | 1381 | 1146 |
| 53 | 1514 | 2625 | 53 | 1379 | 1228 |
| 54 | 1533 | 2829 | 54 | 1384 | 1316 |
| 55 | 1542 | 3047 | 55 | 1405 | 1412 |
| 56 | 1554 | 3277 | 56 | 1410 | 1513 |
| 57 | 1561 | 3520 | 57 | 1420 | 1621 |
| 58 | 1565 | 3779 | 58 | 1424 | 1736 |
| 59 | 1572 | 4054 | 59 | 1444 | 1859 |
| 60 | 1596 | 4348 | 60 | 1473 | 1989 |
| 61 | 1599 | 4659 | 61 | 1477 | 2128 |
| 62 | 1611 | 4988 | 62 | 1498 | 2275 |
| 63 | 1623 | 5337 | 63 | 1511 | 2431 |
| 64 | 1637 | 5708 | 64 | 1535 | 2596 |
| 65 | 1647 | 6101 | 65 | 1549 | 2771 |
| 66 | 1672 | 6517 | 66 | 1563 | 2958 |
| 67 | 1685 | 6957 | 67 | 1570 | 3158 |
| 68 | 1696 | 7423 | 68 | 1587 | 3370 |
| 69 | 1713 | 7919 | 69 | 1597 | 3596 |
| 70 | 1733 | 8445 | 70 | 1611 | 3835 |
| 71 | 1749 | 9006 | 71 | 1629 | 4088 |
| 72 | 1759 | 9602 | 72 | 1648 | 4359 |
| 73 | 1773 | 10236 | 73 | 1667 | 4648 |
| 74 | 1786 | 10909 | 74 | 1677 | 4956 |
| 75 | 1795 | 11628 | 75 | 1693 | 5283 |
| 76 | 1819 | 12398 | 76 | 1704 | 5632 |
| 77 | 1829 | 13222 | 77 | 1718 | 6001 |
| 78 | 1846 | 14109 | 78 | 1728 | 6394 |
| 79 | 1866 | 15067 | 79 | 1750 | 6815 |
| 80 | 1880 | 16105 | 80 | 1771 | 7266 |
| 81 | 1888 | 17232 | 81 | 1785 | 7748 |
| 82 | 1901 | 18458 | 82 | 1806 | 8266 |
| 83 | 1915 | 19802 | 83 | 1830 | 8822 |
| 84 | 1924 | 21277 | 84 | 1846 | 9422 |
| 85 | 1929 | 22907 | 85 | 1872 | 10071 |
| 86 | 1938 | 24723 | 86 | 1890 | 10776 |
| 87 | 1948 | 26782 | 87 | 1910 | 11543 |
| 88 | 1960 | 29134 | 88 | 1931 | 12385 |
| 89 | 1965 | 31854 | 89 | 1956 | 13312 |
| **90** | **1965** | **35046** | **90** | **1984** | **14341** |
| 91 | 1959 | 38823 | 91 | 2016 | 15484 |
| 92 | 1951 | 43358 | 92 | 2052 | 16775 |
| 93 | 1937 | 48867 | 93 | 2082 | 18246 |
| 94 | 1911 | 55643 | 94 | 2126 | 19957 |
| 95 | 1886 | 64102 | 95 | 2176 | 21992 |
| 96 | 1838 | 74790 | 96 | 2253 | 24508 |
| 97 | 1775 | 88635 | 97 | 2370 | 27784 |
| 98 | 1681 | 107588 | 98 | 2632 | 32589 |
| 100 | 1532 | 149827 | 100 | 2967 | 51040 |

**Supplementary Table 9: Fusion gene locus re-annotated by PacBio trascriptome sequencing reads.**

**Supplementary Table 10: The function annotation of novel isoforms using Interproscan.**

**Supplementary Table 11: The locus and types of alternative splicing isoforms by Cufflinks.**

**Supplementary Table 12: The locus and types of alternative splicing isoforms by IDP.**

**Supplementary Table 13: The number of alternative splice isoform with NGS reads (Cufflink) and SMRT reads** (**IDP**)**.**

|  | **A3'S** | **A5'S** | **AE** | **ES** | **IR** | **Total** |
| --- | --- | --- | --- | --- | --- | --- |
| **Num (Cufflink)** | 4912 | 2732 | 1148 | 2040 | 7666 | 18498 |
| **Num (IDP)** | 4940 | 3058 | 955 | 1844 | 9218 | 20015 |

**Supplementary Table 14**: **The summary of five kinds of AS events in root, stem, leaf and rhizome of *P. notoginseng*.**

|  | **Root** | **Stem** | **Leaf** | **Flower** | **Rhizome** |
| --- | --- | --- | --- | --- | --- |
| **A3'S** | 4603 | 3991 | 4103 | 4637 | 4106 |
| **A5'S** | 2474 | 2012 | 2132 | 2474 | 2229 |
| **AE** | 999 | 1144 | 961 | 1197 | 947 |
| **IR** | 5372 | 5113 | 5323 | 6021 | 4543 |
| **ES** | 1636 | 1643 | 1727 | 1895 | 1517 |
| **Total** | 15084 | 13903 | 14246 | 16224 | 13342 |

**Supplementary Table 15**: **The summary of assembled results from the four *Panax* species.**

|  | ***P. ginseng*** | ***P. quinquefolium*** | ***P. vietnamensis*** | ***P. japonicus*** |
| --- | --- | --- | --- | --- |
| **Total unigenes** | 185,759 | 62,237 | 66,524 | 113,959 |
| **Total transcripts:** | 267,571 | 131,561 | 121,389 | 202,053 |
| **Total residues** | 185,375,939 | 94,410,636 | 120,184,658 | 151,697,909 |
| **Smallest transcript:** | 201 | 201 | 201 | 201 |
| **Largest transcript:** | 11,466 | 8,013 | 12,133 | 11,588 |
| **Transcript N50** | 1,071 | 1,028 | 1,656 | 1,163 |
| **Unigene N50** | 726 | 866 | 1,201 | 954 |
| **Average length:** | 693 | 718 | 990 | 751 |

**Supplementary Table 16**: **The CDS prediction of the four *Panax* species.**

|  | ***P. ginseng*** | ***P. quinquefolium*** | ***P. vietnamensis*** | | ***P. japonicus*** |
| --- | --- | --- | --- | --- | --- |
| **Number** | 54,081 | 26,751 | 22,406 | 47,364 | |

**Supplementary Table 17**: **The clustering of gene families from *P. notoginseng*, other four *Panax* species and *Daucus carota*.**

| **Species** | **Genes** | **Unclustered genes** | **Clustered genes** | **Families** | **Unique families** | **Unique families**  **genes** | **Common families** | **Common families genes** | **Single copy** | **Average genes per family** |
| --- | --- | --- | --- | --- | --- | --- | --- | --- | --- | --- |
| *Daucus carota* | 44,655 | 3,175 | 41,480 | 14,685 | 1,542 | 6,346 | 8,074 | 24,233 | 1,416 | 2.825 |
| *P. notoginseng* | 48,314 | 12,455 | 35,859 | 15,132 | 1,847 | 5,965 | 8,074 | 22,334 | 1,416 | 2.37 |
| *P. ginseng* | 54,081 | 23,392 | 30,689 | 21,137 | 2,100 | 4,852 | 8,074 | 13,503 | 1,416 | 1.452 |
| *P. quinquefolium* | 26,751 | 6,281 | 20,470 | 16,321 | 34 | 81 | 8,074 | 11,717 | 1,416 | 1.254 |
| *P. vietnamensis* | 22,406 | 4,150 | 18,256 | 15,509 | 15 | 33 | 8,074 | 10,498 | 1,416 | 1.177 |
| *P. japonicus* | 47,364 | 20,346 | 27,018 | 18,183 | 629 | 1,630 | 8,074 | 14,957 | 1,416 | 1.486 |

**Supplementary Table 18**: **The genes are involved the ginsenoside biosynthesis of *P. notoginseng*.**

| **Enzyme name** | **EC number*** | **Abbreviation** | **Copy number*** | |
| --- | --- | --- | --- | --- |
| Acetyl-CoA acetyltransferase | 2.3.1.9 | ACAT | 10 |  |
| Hydroxymethyl glutaryl CoA synthase | 2.3.3.10 | HMGS | 57 |  |
| 3-hydroxy-3-methylglutaryl-coenzymeA reductase | 1.1.1.34 | HMGR | 8 |  |
| Mevalonate kinase | 2.7.1.36 | MK | 9 |  |
| Phosphomevalonate kinase | 2.7.4.2 | PMK | 2 |  |
| Mevalonate diphosphosphate decarboxylase | 4.1.1.33 | MDD | 4 |  |
| 1-deoxy-D-xylulose-5-phosphate synthase | 2.2.1.7 | DXS | 20 |  |
| 1-deoxy-D-xylulose-5-phosphate reductoisomerase | 1.1.1.267 | DXR | 11 |  |
| 2-C-methyl-D-erythritol 4-phosphate cytidylyltransferase | 2.7.7.60 | MCT | 3 |  |
| 4-diphosphocytidyl-2-C-methyl-D-erythritol kinase | 2.7.1.148 | CMK | 2 |  |
| 2-C-methyl-D-erythritol 2,4-cyclodiphosphate synthase | 4.6.1.12 | MDS | 2 |  |
| (E)-4-hydroxy-3-methylbut-2-enyl-diphosphate synthase | 1.17.7.1/3 | HDS | 14 |  |
| 4-hydroxy-3-methylbut-2-en-1-yl diphosphate reductase | 1.17.7.4 | HDR | 4 |  |
| Isopentenylpyrophosphate isomerase | 5.3.3.2 | IPI | 3 |  |
| Geranylgeranyl pyrophosphate synthase | 2.5.1.29 | GGPS | 5 |  |
| Geranylgeranyl diphosphate synthase | 2.5.1.1 | GGR | 5 |  |
| Farnesyl diphosphate synthase | 2.5.1.10 | FPS | 9 |  |
| Squalene synthase | 2.5.1.21 | SS | 6 |  |
| Squalene epoxidase | 1.14.14.17 | SE | 22 |  |
| β-amyrin synthase | 5.4.99.39 | AS | 10 |  |
| Dammarenediol-II synthase | 4.2.1.125 | DDS | 14 |  |
| Cytochrome P450 |  | CYP450 | 170 |  |
| Gycosyl transferase  * EC number: [Enzyme Commission number](https://en.wikipedia.org/wiki/Enzyme_Commission_number);  * Copy number: The number of unigenes to encode relevant enzymes. |  | GT | 189 |  |

**Supplementary Table 19**. **Summary of RNA sequencing (RNA-Seq) of different developed time and different tissues of *P. notoginseng*.**

| **Tissues** | **Read length (bp)** | **No. of reads (M*)** | **Clean data (Gb)** |
| --- | --- | --- | --- |
| **One-year old Root Reapet1** | 100 | ~33.6 | ~4.2 |
| **One-year old Stem Reapet1** | 100 | ~32.3 | ~4.1 |
| **One-year old Leaf Reapet1** | 100 | ~44.2 | ~5.5 |
| **One-year old Root Reapet2** | 100 | ~40.7 | ~5.1 |
| **One-year old Stem Reapet2** | 100 | ~32.9 | ~4.1 |
| **One-year old Leaf Reapet2** | 100 | ~37.1 | ~4.7 |
| **One-year old Root Reapet3** | 100 | ~37.6 | ~4.7 |
| **One-year old Stem Reapet3** | 100 | ~34.6 | ~4.2 |
| **One-year old Leaf Reapet3** | 100 | ~39.2 | ~4.9 |
| **Two-year old Root Reapet1** | 100 | ~48.4 | ~6.1 |
| **Two-year old Stem Reapet1** | 100 | ~33.8 | ~4.2 |
| **Two-year old Leaf Reapet1** | 100 | ~40.1 | ~5.0 |
| **Two-year old Flower Reapet1** | 100 | ~38.2 | ~4.8 |
| **Two-year old Root Reapet2** | 100 | ~44.2 | ~5.5 |
| **Two-year old Stem Reapet2** | 100 | ~40.8 | ~5.1 |
| **Two-year old Leaf Reapet2** | 100 | ~34.0 | ~4.3 |
| **Two-year old Flower Reapet2** | 100 | ~30.5 | ~3.8 |
| **Two-year old Root Reapet3** | 100 | ~48.7 | ~6.1 |
| **Two-year old Stem Reapet3** | 100 | ~35.5 | ~4.5 |
| **Two-year old Leaf Reapet3** | 100 | ~34.5 | ~4.3 |
| **Two-year old Flower Reapet3** | 100 | ~41.1 | ~5.1 |
| **Three-year old Root Reapet1** | 100 | ~44.3 | ~5.5 |
| **Three -year old Stem Reapet1** | 100 | ~44.0 | ~5.5 |
| **Three -year old Leaf Reapet1** | 100 | ~47.5 | ~5.9 |
| **Three-year old Flower Reapet1** | 100 | ~39.1 | ~4.9 |
| **Three-year old Root Reapet2** | 100 | ~47.8 | ~6.0 |
| **Three -year old Stem Reapet2** | 100 | ~34.3 | ~4.2 |
| **Three -year old Leaf Reapet2** | 100 | ~34.1 | ~4.3 |
| **Three-year old Flower Reapet2** | 100 | ~38.9 | ~4.9 |
| **Three-year old Root Reapet3** | 100 | ~49.3 | ~6.2 |
| **Three -year old Stem Reapet3** | 100 | ~41.4 | ~5.2 |
| **Three -year old Leaf Reapet3** | 100 | ~42.0 | ~5.2 |
| **Three-year old Flower Reapet3** | 100 | ~42.2 | ~5.3 |
| **Total** |  | **1306.9** | **158.3** |

**Supplementary Table 20: The summary of paired-end reads mapped to the reference transcriptome of *P. notoginseng***.

| **Sample** | **Total reads** | **Mapping ratio** | **Unique mapping**  **(bp)** | **Unique mapping ratio** | **Multiple mapping**  **(bp)** | **Multiple mapping ratio** |
| --- | --- | --- | --- | --- | --- | --- |
| S61-T100 | 23,912,363 | 76.39% | 6,013,967 | 25.15% | 12,251,631 | 51.24% |
| S61-T102 | 24,367,552 | 72.67% | 5,969,560 | 24.50% | 11,738,067 | 48.17% |
| S61-T103 | 24,672,745 | 76.85% | 6,384,406 | 25.88% | 12,576,891 | 50.97% |
| S61-T20 | 16,260,805 | 77.81% | 4,199,686 | 25.83% | 8,453,585 | 51.99% |
| S61-T21 | 16,890,171 | 78.19% | 4,392,418 | 26.01% | 8,814,374 | 52.19% |
| S61-T22 | 16,477,393 | 77.08% | 4,292,360 | 26.05% | 8,407,968 | 51.03% |
| S61-T23 | 20,369,634 | 77.59% | 5,234,007 | 25.70% | 10,570,887 | 51.90% |
| S61-T24 | 19,587,072 | 77.02% | 4,247,254 | 21.68% | 10,838,704 | 55.34% |
| S61-T25 | 17,308,898 | 75.83% | 4,442,460 | 25.67% | 8,683,738 | 50.17% |
| S61-T26 | 18,815,639 | 77.00% | 4,725,985 | 25.12% | 9,762,505 | 51.89% |
| S61-T28 | 16,904,984 | 77.84% | 4,434,100 | 26.23% | 8,725,156 | 51.61% |
| S61-T29 | 19,115,210 | 72.37% | 4,898,652 | 25.63% | 8,934,267 | 46.74% |
| S61-T30 | 20,401,211 | 76.78% | 5,305,876 | 26.01% | 10,358,099 | 50.77% |
| S61-T31 | 16,992,668 | 77.89% | 4,213,500 | 24.80% | 9,021,477 | 53.09% |
| S61-T32 | 15,256,954 | 68.13% | 3,650,366 | 23.93% | 6,743,633 | 44.20% |
| S61-T33 | 17,769,471 | 77.42% | 4,468,087 | 25.14% | 9,289,478 | 52.28% |
| S61-T34 | 17,247,500 | 75.93% | 4,211,495 | 24.42% | 8,884,291 | 51.51% |
| S61-T35 | 20,543,840 | 72.77% | 5,156,082 | 25.10% | 9,794,369 | 47.68% |
| S61-T36 | 22,006,139 | 77.14% | 5,593,261 | 25.42% | 11,381,631 | 51.72% |
| S61-T37 | 23,751,512 | 76.66% | 5,780,879 | 24.34% | 12,427,454 | 52.32% |
| S61-T38 | 19,591,817 | 75.15% | 4,820,897 | 24.61% | 9,901,758 | 50.54% |
| S61-T41 | 17,145,770 | 76.57% | 4,273,809 | 24.93% | 8,855,364 | 51.65% |
| S61-T42 | 17,060,994 | 76.73% | 4,348,238 | 25.49% | 8,742,061 | 51.24% |
| S61-T43 | 19,463,827 | 73.47% | 4,916,266 | 25.26% | 9,382,986 | 48.21% |
| S61-T44 | 20,725,605 | 76.82% | 5,384,960 | 25.98% | 10,535,519 | 50.83% |
| S61-T45 | 21,164,535 | 74.18% | 5,359,873 | 25.32% | 10,338,968 | 48.85% |
| S61-T52 | 22,149,049 | 77.18% | 5,620,340 | 25.38% | 11,474,022 | 51.80% |
| S61-T93 | 20,059,187 | 76.93% | 5,115,983 | 25.50% | 10,316,482 | 51.43% |
| S61-T95 | 21,012,299 | 63.56% | 3,977,626 | 18.93% | 9,377,613 | 44.63% |
| S61-T96 | 22,089,180 | 75.57% | 5,509,827 | 24.94% | 11,182,479 | 50.62% |
| S61-T97 | 22,160,343 | 76.60% | 6,273,493 | 28.31% | 10,701,009 | 48.29% |
| S61-T98 | 18,566,875 | 75.80% | 4,587,006 | 24.71% | 9,486,336 | 51.09% |
| S61-T99 | 24,204,978 | 75.18% | 5,974,888 | 24.68% | 12,223,372 | 50.50% |

**Supplementary Table 21**: **Levels of gene expression (TPM) of all transcripts in 11 different tissues with the three biological replicates in *P. notoginseng*.**

**Supplementary Table 22**: **Statistical tests of differential expression of genes involved the ginsenoside biosynthesis with the three biological replicates in *P. notoginseng*.**

**Supplementary Table 23**: **Copy number variation of genes involved in the ginsenoside biosynthesis in *P. notoginseng*, *D.carota* and four other *Panax* species**

|  | ***P. notoginseng*** | ***D. carota*** | ***P. ginseng*** | ***P. quinquefolius*** | ***P. vietnamensis*** | ***P. japonicus*** |
| --- | --- | --- | --- | --- | --- | --- |
| *ACAT* | 10 | 5 | 4 | 2 | 2 | 3 |
| *AS* | 10 | 6 | 1 | 2 | 0 | 1 |
| *CMK* | 2 | 1 | 3 | 1 | 1 | 2 |
| *DDS* | 14 | 3 | 1 | 1 | 1 | 1 |
| *DXR* | 11 | 1 | 2 | 1 | 1 | 1 |
| *DXS* | 20 | 5 | 6 | 3 | 3 | 7 |
| *FPS* | 9 | 1 | 9 | 3 | 1 | 7 |
| *GGPS_GGR* | 5 | 8 | 6 | 2 | 3 | 13 |
| *GPS* | 0 | 4 | 3 | 1 | 1 | 4 |
| *HDR* | 4 | 4 | 3 | 1 | 3 | 2 |
| *HDS* | 14 | 5 | 2 | 1 | 1 | 4 |
| *HMGR* | 8 | 3 | 5 | 4 | 2 | 10 |
| *HMGS* | 57 | 1 | 3 | 2 | 1 | 3 |
| *IPI* | 3 | 3 | 5 | 0 | 2 | 3 |
| *MCT* | 3 | 3 | 3 | 1 | 1 | 2 |
| *MDD* | 4 | 1 | 1 | 1 | 1 | 2 |
| *MDS* | 2 | 1 | 2 | 1 | 2 | 2 |
| *MK* | 9 | 2 | 1 | 1 | 1 | 1 |
| *PMK* | 2 | 3 | 2 | 3 | 1 | 1 |
| *SE* | 22 | 5 | 8 | 8 | 3 | 5 |
| *SS* | 6 | 2 | 2 | 1 | 1 | 4 |

**Supplementary Table 24: The chemical formulae of ginsenosides.**

| **Ginsenoside** | **Chemical formulae** |
| --- | --- |
| **Rg1** |  |
| **Rb1** |  |
| **Rd** |  |
| **Re** |  |
| **Rh1** |  |
| **R1** |  |
| **Rb2** |  |
| **Rg3** |  |
| **F1** |  |
| **F2** |  |
| **CK** |  |
| **Fa** |  |
| **Fc** |  |
| **Fe** |  |

**Supplementary Table S25: The seventeen characteristic compounds in different tissues from 1-year, 2-yearand 3-year *P. notoginseng* plants detected using High Performance Liquid Chromatography (HPLC).**
